# Alternating Current Stimulation for Vision Restoration after Optic Nerve Damage: A Randomized Clinical Trial

**DOI:** 10.1371/journal.pone.0156134

**Published:** 2016-06-29

**Authors:** Carolin Gall, Sein Schmidt, Michael P. Schittkowski, Andrea Antal, Géza Gergely Ambrus, Walter Paulus, Moritz Dannhauer, Romualda Michalik, Alf Mante, Michal Bola, Anke Lux, Siegfried Kropf, Stephan A. Brandt, Bernhard A. Sabel

**Affiliations:** 1 Institute of Medical Psychology, Medical Faculty, Otto-von-Guericke University Magdeburg, Magdeburg, Germany; 2 Department of Neurology, Charité-Universitätsmedizin Berlin, Berlin, Germany; 3 Department of Ophthalmology, University Medical Center, Georg-August University of Goettingen, Goettingen, Germany; 4 Department of Clinical Neurophysiology, University Medical Center, Georg-August University, Goettingen, Germany; 5 Center for Integrative Biomedical Computing and the Scientific Computing and Imaging Institute, University of Utah, Salt Lake City, Utah, United States of America; 6 Institute for Biometry and Medical Informatics, Otto-von-Guericke University Magdeburg, Magdeburg, Germany; University of Utah, UNITED STATES

## Abstract

**Background:**

Vision loss after optic neuropathy is considered irreversible. Here, repetitive transorbital alternating current stimulation (rtACS) was applied in partially blind patients with the goal of activating their residual vision.

**Methods:**

We conducted a multicenter, prospective, randomized, double-blind, sham-controlled trial in an ambulatory setting with daily application of rtACS (n = 45) or sham-stimulation (n = 37) for 50 min for a duration of 10 week days. A volunteer sample of patients with optic nerve damage (mean age 59.1 yrs) was recruited. The primary outcome measure for efficacy was super-threshold visual fields with 48 hrs after the last treatment day and at 2-months follow-up. Secondary outcome measures were near-threshold visual fields, reaction time, visual acuity, and resting-state EEGs to assess changes in brain physiology.

**Results:**

The rtACS-treated group had a mean improvement in visual field of 24.0% which was significantly greater than after sham-stimulation (2.5%). This improvement persisted for at least 2 months in terms of both within- and between-group comparisons. Secondary analyses revealed improvements of near-threshold visual fields in the central 5° and increased thresholds in static perimetry after rtACS and improved reaction times, but visual acuity did not change compared to shams. Visual field improvement induced by rtACS was associated with EEG power-spectra and coherence alterations in visual cortical networks which are interpreted as signs of neuromodulation. Current flow simulation indicates current in the frontal cortex, eye, and optic nerve and in the subcortical but not in the cortical regions.

**Conclusion:**

rtACS treatment is a safe and effective means to partially restore vision after optic nerve damage probably by modulating brain plasticity. This class 1 evidence suggests that visual fields can be improved in a clinically meaningful way.

**Trial Registration:**

ClinicalTrials.gov NCT01280877

## Introduction

Optic neuropathy and glaucoma are frequent causes of chronic blindness [1]. After a spontaneous recovery period, visual field (VF) loss is considered irreversible which compromises vision-related quality of life (QoL) [2–4]. A systematic search for a means to improve such vision loss is urgently needed.

Transcranial direct current stimulation (tDCS) and alternating current stimulation (ACS) of the brain are new methods to modify brain excitability and synchronicity in healthy subjects and patients with brain injuries affecting different modalities such as the motor [5], language [6], or somatosensory domains [7]. In the visual system, exploratory evidence indicates that ACS can enhance neuronal function both in normal subjects [8–9] and in patients with VF loss who still have some residual vision [10]. The proposed mechanism of action is neuromodulation of oscillatory brain activity towards a more synchronized EEG via entrainment of specific stimulation frequencies [10–11], and the reorganization of brain functional connectivity networks [12] is considered to be a novel and promising avenue for visual rehabilitation [13].

In a small sample study, ACS was delivered in frequencies ranging from theta to high beta via electrodes placed near the eye to re-activate residual vision in optic neuropathy [14]. Here, 10 daily rtACS sessions increased light detection performance and improved patient-reported vision-related QoL which was moderately correlated with VF gains [15]. These improvements were associated with increased alpha-power at occipital sites in resting-EEGs [14,16] and increased neuronal synchronization of functional connectivities between the occipital and frontal regions [12]. Yet, the level of clinical evidence is still not definitive because the trials included only small patient samples [12,14–16]. We hypothesized that efficacy and safety could be documented in a larger sample prospective trial.

We now report the first confirmatory, large-sample, double blind, randomized, multi-center clinical trial to establish the efficacy and safety of rtACS stimulation in patients with vision impairments caused by optic nerve damage.

## Methods

### Study design

**Fig 1:**
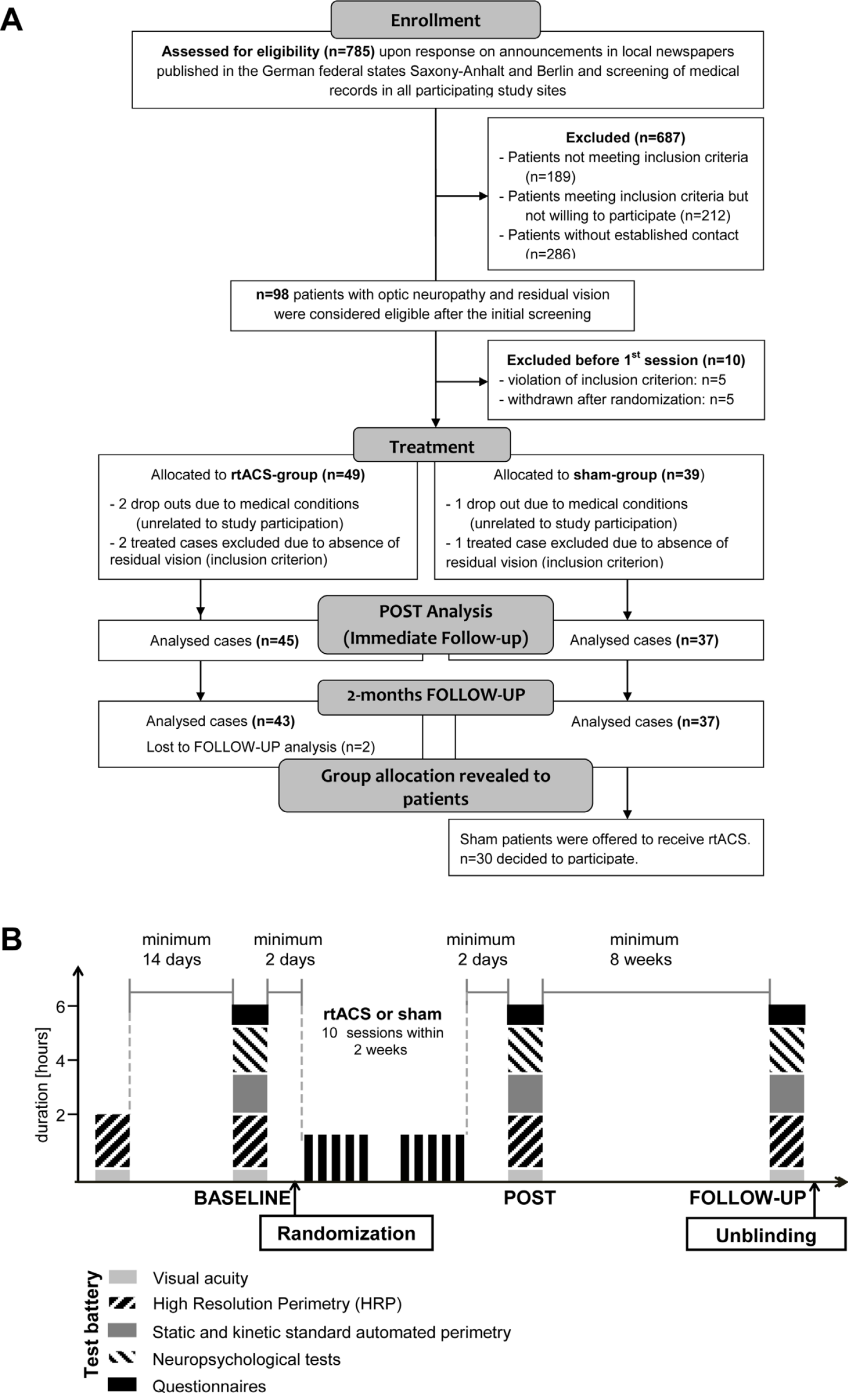
Consort flow chart and study design. **(A)** Patient flow for cases included in the primary outcome measure analysis. Of 98 eligible patients, 45 were treated with rtACS and 37 with sham-stimulation. Five subjects left the study between initial screening and BASELINE for different reasons and another five subjects were excluded due to violation of an inclusion criterion (unacceptable fluctuations between initial screening and BASELINE). During the treatment phase three subjects dropped out because of medical conditions that were unrelated to study participation. Three treated cases of legally blind subjects were excluded from subsequent analyses due to violation of inclusion criterion (no residual vision). **(B)** Study design with diagnostic and treatment visits. Randomization was done after BASELINE assessment. Stability of VF defects was ascertained by comparing VFs at BASELINE with those obtained during the screening visit 2 weeks earlier. Upon completion of the 10-day treatment, all initial diagnostic tests were repeated (POST). The FOLLOW-UP diagnostic assessment was conducted after a therapy-free interval of at least 2 months.

The trial flow diagram and study design are shown in Fig 1A and 1B. Diagnostic evaluations were conducted at BASELINE, after completion of the 10-day treatment (POST), and after a 2-month treatment-free interval (FOLLOW-UP). The CONSORT checklist (S1 File) and statistical analyses protocol (S2 File) are included as supporting information.

The study design was prospective and double blind; neither the patients nor the diagnostic examiners were aware to which treatment arm the patient belonged. For allocation concealment, patients of the sham-group received minimal dose stimulation. The investigator administering the treatment was aware of the group identity but was instructed not to reveal to the patients which treatment they received. Patients were informed to which group they belonged only after the FOLLOW-UP tests, and all sham-stimulation patients were offered rtACS. Informed written consent was obtained prior to randomization. Study participants received a compensation for study participation. The ethics committees of the participating study centers in Magdeburg, Berlin, and Goettingen approved the study, and a statistical analysis plan was filed with the lead ethics committee. The study was conducted according to the Declaration of Helsinki and registered at ClinicalTrials.gov (NCT01280877). The trial was registered in January 2011 upon receiving approval from the leading ethics committee in December 2010. The date range for patient recruitment and follow-up was from December 2010 until February 2012. The authors confirm that all ongoing and related trials for this intervention are registered.

### Study sample description

Patients who suffered visual field loss caused by glaucoma (n = 33) or AION (n = 32) were randomized. The following etiologies of optic atrophy were also included: post-acute inflammation (n = 12), optic nerve compression (n = 5, of which 4 were tumor-induced and 1 intracranial hemorrhage), congenital or unknown etiology of optic atrophy (n = 5), and Leber's hereditary optic neuropathy (n = 3). Eight patients had two concomitant diagnoses of optic nerve atrophy. The sample sizes at each study center were as follows: Charité Berlin (n = 33), Otto-von-Guericke University Magdeburg (n = 31), and Department of Ophthalmology, Georg-August University of Goettingen/ Eye Clinic Kassel (n = 18). Table 1 summarizes the demographics and lesion variables. With respect to BASELINE variables, results did not significantly differ between the rtACS- and the sham-group (Table 2).

**Table 1:** Patients’ demographics and lesion characteristics.

		rtACS-group	Sham-group	p
Sample size, n		45	37	
Age (years), mean ± standard error mean (SEM)		57.8 ± 14.2	60.7 ± 11.6	0.563
Male, (%)		71.1	40.5	0.005
Lesion age,	6–12 months (%)	13.9	7.4	0.206
right eye	1–2 years (%)	8.3	0.0	
	> 2 years (%)	77.8	92.6	
Lesion age,	6–12 months (%)	8.3	14.8	0.468
left eye	1–2 years (%)	16.7	7.4	
	> 2 years (%)	75.0	77.8	
single diagnosis		88.9	89.2	0.965
dual diagnosis		11.1	10.8	
Binocular lesions n(%)^1^		60.0	46.0	0.442
Monocular lesions, one eye intact (%)		31.1	45.9	
Monocular lesions, one eye blind (%)		8.9	8.1	

^1^ In cases with binocular vision loss both eyes were averaged for subsequent analyses. P-values are reported for Wilcoxon-Mann-Whitney U tests, two-sided and Pearson-Chi-Square tests, two-sided.

**Table 2:** Visual field characteristics at BASELINE according to treatment arms.

		rtACS-group	Sham-group	p
**High-resolution perimetry**				
	Detection accuracy whole VF (%)	44.59 [25.18; 63.82]	53.48 [37.34; 75.70]	0.142
	Detection accuracy defective VF sectors (%)^1^	17.92 [12.06; 31.03]	23.31 [14.37; 38.94]	0.228
	Detection accuracy within 5° VF (%)	56.25 [34.38; 75.35]	62.50 [45.83; 76.74]	0.459
	Fixation accuracy (%)	91.70 [83.46; 97.26]	94.25 [84.40; 97.51]	0.586
	False positive reactions (%)	2.18 [0.85; 4.00]	1.30 [0.49; 3.57]	0.155
	Reaction time whole VF (ms)	525 [461; 575]	509 [463; 569]	0.394
	Reaction time HRP defective VF sectors (ms)	554 [510; 581]	544 [485; 575]	0.261
	Reaction time within 5° VF (ms)	484 [432; 565]	480 [429; 519]	0.554
**Standard automated perimetry**				
	Foveal threshold, static perimetry (dB)	21.25 [15.50; 27.00]	25.00 [19.00; 28.50]	0.113
	Mean threshold, static perimetry (whole VF, dB)	8.78 [5.91; 15.74]	11.95 [6.7; 15.97]	0.320
	Fixation accuracy, static perimetry (%)	93.81 [69.25; 100]	94.87 [86.13; 100]	0.259
	Mean eccentricity, kinetic perimetry (degree)	46.82 [34.29; 55.40]	48.13 [28.09; 56.38]	0.899
	Mean VF size, kinetic perimetry (square degree)	7280 [4461; 9743]	7907 [3131; 10053]	0.817
**Visual acuity (logMAR)**^2^				
	Uncorrected near vision (n = 77)	0.75 [0.45; 1.10]	0.80 [0.60; 1.10]	0.267
	Uncorrected far vision (n = 69)	0.50 [0.29; 0.92]	0.44 [0.22; 0.70]	0.708

Results are given as medians and interquartile ranges. The groups did not differ significantly in any of the BASELINE measures (Wilcoxon-Mann-Whitney U tests, two-sided).

^1^ To balance the groups for randomization with respect to defect depth, the BASELINE VF defect was classified as having a high or low defect depth with a threshold of 30% detection accuracy in the defective VF. Based on this classification, 55 patients (67.1%) belonged to the high and 27 (32.9%) to the low defect depth group. Between-group differences of the BASELINE diagnostic values were not statistically significant in any measure (Wilcoxon-Mann-Whitney U test, two-sided).

^2^ Visual acuity was calculated for all patients with better visual acuity than counting fingers (logMAR = 3). Therefore, five subjects had to be excluded from the recording of near vision and 13 subjects from far vision acuity.

### Sample size and randomization

The sample size calculation was based on results of two earlier pilot studies at the Institute of Medical Psychology (University of Magdeburg) considering similar patients and equivalent stimulation schemes, i.e., rtACS- and sham-groups as in the present trial. In the first trial a mean percentage increase (± standard deviation) of 65.61 ± 104.35 was observed in the rtACS-group (n = 19) and 16.93 ± 31.22 in the sham-group (n = 14). The corresponding results of the subsequent trial were 30.68 ± 41.97 in the stimulation group (n = 12) and 9.57 ± 12.05 (n = 13) for the sham group. Using these means and standard deviations in a sample size calculation (α = 0.05, two-sided, power 1 – β = 0.80) for a two-sample t-test (Satterthwaite version) with nQuery Advisor 7.0 resulted in 41 patients per group for the first trial and 36 patients per group for the second trial. Therefore, we adopted a conservative approach of considering up to 10 percent drop-out and planned to recruit 45 patients per group. Patients were assigned to either the rtACS- or the sham-group by “Randomization In Treatment Arms” software (RITA, StatSol, Lübeck) with stratified block randomization considering the study center and the VF defect depth at BASELINE as potential prognostic factors. High vs. low defect depth was defined as stimulus detection rates below vs. above 30% inside the VF defect.

Randomization resulted in comparable average baseline situation and demographics in the rtACS- and sham-group. Only the percentage of males vs. females was unequally distributed but this was independent of study results.

### Inclusion and exclusion criteria

The following criteria were established prospectively. Inclusion criteria included: (i) stable VF defect with residual vision as detected by super-threshold perimetry testing (HRP, cut-off 1.5% in at least one eye); (ii) lesion age >6 months; (iii) patient age >18 yrs; (iv) compliance with the experimenters’ instructions during diagnostic testing; and (v) sufficient fixation ability. Exclusion criteria included: electric or electronic implants (such as cardiac pacemaker); any metal artifacts in the head or truncus area (with the exception of dental implants); epilepsy and photo-sensibility; acute auto-immune diseases; psychiatric diagnoses; diabetic retinopathy or other documented retinal impairments; high blood pressure (>160/100 mmHg); acute conjunctivitis; retinitis pigmentosa; pathological nystagmus; an unstable or high level of intraocular pressure (> 27 mm Hg); and presence of an un-operated tumor or cancer recurrence anywhere in the body.

### Statistical analyses and study endpoints

Primary data analysis was conducted independently by the biometry department of two of the coauthors (A.L., S.K.) and included sample size calculation, statistical analysis plan, initialization of randomization and data source verification. Primary and secondary endpoints were defined prospectively.

VF mapping was conducted using a campimetric, high-resolution, super-threshold visual detection test (HRP) [17], a method that reveals areas of residual vision in the central VF with lower inter-test-variability than standard static perimetry due to target luminance above threshold. In a darkened room, the patient was viewing a 17" monitor from a chin-head-rest at a distance of 42 cm. White target stimuli were presented at random in a grid of 25x19 stimulus locations and the task was to hit the space bar. The procedure included a fixation control using isoluminant color changes of the fixation point. Eyes with intact vision or complete blindness were not considered. The primary endpoint was “percent change over BASELINE” in the HRP detection rate.

Secondary endpoints of HRP were percentage change of the stimulus detection rate in the defective VF (excluding intact areas), the central 5°-VF, and average reaction time in total HRP VF. Further secondary endpoints were static and kinetic perimetry results (foveal and mean threshold of 30°-VFs, mean eccentricity/VF size), best corrected near and far visual acuity (Landolt), and patient-reported outcome, i.e., an intervention-related questionnaire with a structured response format and a vision-related QoL questionnaire (NEI-VFQ-39) [18] to evaluate subjective improvements of “visual field defect and related impairments” (15 items) and “general health and mental distress” (four items) at POST and FOLLOW-UP. Concerning static perimetry foveal threshold and mean threshold of 30°-VFs were obtained. A fast threshold strategy was used to determine threshold values at 66 positions within the 30° visual field. Target stimuli (size: III/4mm^2^, white, luminance: 318 cd/m^2^/ 0db, duration: 0.2 sec) were presented on a background with constant luminance of 10cd/m2. Mean threshold refers to the average dB values of 66 test positions. In kinetic perimetry the VF border was determined for 24 meridians (spaced by 15°) in randomized order at a constant luminance threshold of III/4e (0dB) and a velocity of 2° per sec. Mean eccentricity refers to the average of 24 meridians. Mean visual field size refers to the area inside the VF border in kinetic perimetry, i.e., the sum of 24 triangles (X axis eccentricity multiplied by Y axis eccentricity /2).

The percent change over BASELINE was determined at 48 hrs (minimum interval) after the last stimulation day (i.e., the 10th stimulation session) (POST) and at 2 months FOLLOW-UP (FU) for each endpoint as 100*(POST resp. FOLLOW-UP–BASELINE) / BASELINE. HRP reaction time (absolute differences), visual acuity (logarithmic values) and questionnaire data were shown as absolute values. For binocular defects the value of both eyes was averaged. Since there were no center effects (neither as the main factor nor as an interaction with the treatment arm), nor dependencies of BASELINE results for the primary endpoint, the primary data analysis and secondary between-group comparisons were performed for the pre-defined hypothesis with a one-sided U-test (p-value<0.05). The Hodges-Lehmann effect estimator and 95%-confidence intervals (CI) are reported. Within-group comparisons were calculated with Wilcoxon matched-pairs signed rank-tests. Analyses were done in MatlabR2011b and SPSS 21.

### EEG power spectra after rtACS

All subjects were seated in a dimly lit room and instructed to keep their eyes closed during the whole recording session. Electroencephalography (EEG) was recorded for 2 min before and after each stimulation session using 16 electrodes (10–20 system) with impedances <10kΩ with eyes closed (at rest) in a darkened room. The EEG-signal at occipital (O1, O2) areas of interest (AOIs) was referenced against the grand-average and preprocessed with ASA™- software (ANT, Enschede, Netherlands) including a bandpass-filter (0.5–40Hz, filter-slope 24dB/oct.), 2 sec bins, and baseline correction. Power-spectra at occipital sites O1 and O2 were assessed with Fast Fourier Transformation (FFT) to determine bandwidth specific power (μV2) with automatic artifact-rejection (min/max allowed amplitude: ±75.00μV), normalized power-spectra and DC-correction during FFT-averaging. Resting state eyes-closed EEG immediately before and after one day of treatment was analyzed after the first rtACS session. The EEG data were analyzed in 3 steps. First, we defined 5 spectral bands: delta, 1–3 Hz; theta, 3–7 Hz; alpha (alpha I), 7–14 Hz; and beta, 14–30 Hz. Between-group comparisons were conducted with independent samples t test. The effects of rtACS or sham-stimulation on EEG measures were analyzed as ΔEEG = EEGpost−EEGpre. To assess functional interactions between brain regions coherence was analyzed indicating coupling between two signals as a function of frequency [19]. Coherence was calculated for each pair of channels *ij* and defined as follows:

$$C_{ij}(f)=\frac{{\left|S_{ij}(f)\right|}^{2}}{S_{ii}(f)\;S_{jj}(f)}$$

In this equation *S* denotes the spectrum of signals from two EEG channels *i* and *j*, for a given frequency bin *f*.

Changes in spectral power of oscillatory brain activity and strength of functional connectivity over the visual cortex after the first stimulation session were then related to the primary outcome measure. To assess the relationship between EEG and primary outcome measures, Spearman correlation coefficient was used.

### Repetitive transorbital alternating current stimulation

After BASELINE assessments patients were stimulated with either rtACS or sham-stimulation on 2 × 5 weekdays for 25 min at day1 to 50 min at day10 with eyes closed. Four 10mm Grass gold electrodes (SAFELEAD™, Astro-Med, Inc, USA), 2 for each eye, were placed on the skin near the orbital cavity (“transorbital”), and biphasic square-pulses were applied in bursts (Alpha SYNC stimulator, EBS Germany). The return electrode (stainless steel plate, 32x30 mm) was positioned on the right arm. Stimulation current strength was 125% of phosphene threshold recorded during 5 Hz stimulation. rtACS was conducted with frequencies between 8–25 Hz. To conceal group identity, sham-patients received only one ACS burst per minute (“minimal” stimulation) to create phosphene experiences.

### Safety

Possible side effects were evaluated by a semi-structured daily interview that included queries on mild headache, discomfort, and vertigo as expected minor adverse events. Patients were also asked at the end of each daily stimulation session and at POST and FOLLOW-UP to report any adverse effects, such as uncommon or uncomfortable sensations.

### Simulating alternating current flow

For assessing current flow properties of rtACS, a single computer simulation was performed to mimic stimulation using an electrode placed over the right eye and return electrode at the neck region of the subject. Although four electrodes were used for treatment, they were only used one at a time. Therefore, the current flow simulation was done only with one electrode, representing all other electrodes. A finite element model was developed using multi-modal imaging data (MRI/CT) of a 40-yrs-old male. Isotropic conductivities were assigned to head tissues such as scalp, cerebrospinal fluid, gray and white matter (0.43, 1.79, 0.33, 0.142; [20]), air pockets (1e-6 S/m), skull (0.01 S/m [21–22], and eye tissue (0.6, 1.05, 0.4 S/m for sclera, intraocular and nerve tissue [23]. One circular electrode (height/diameter: 3/10 mm, conductivity: 1.5 S/m) was placed above the subject's right eyebrow and a return electrode was modeled as the last axial slice of the neck region (SCIRun software, current injection: +/-0.5 mA [21]. The simulation represents a quasi-static solution of the Maxwell equations and serves as an approximation for current density estimation of one time sample during rtACS when the cathode and anode reaches its maximal current intensity value.

## Results

### Safety

None of the participants reported discomfort during the stimulation. One serious adverse event with subsequent hospitalization occurred, but it was unrelated to rtACS. Transient vertigo was reported by two rtACS-subjects in a total of five sessions and one sham-subject experienced persistent vertigo for 0.5 hrs after each session. Temporary dizziness was reported once after a single rtACS-session. Mild headache during a single stimulation session was reported once by an rtACS-subject and once by a sham-subject. Further reports were mild headaches immediately after a stimulation session by four rtACS- and two sham-subjects and cutaneous sensations in eight sham- and 12 rtACS-subjects. Back pain and stiff neck were reported by one rtACS-subject on the first four stimulation days. This subject dropped out of the study.

Concerning autonomic activity, we did not observe meaningful changes induced by rtACS (pre-session rtACS values: systolic pressure 131.54±1.94mmHG, diastolic pressure 82.81 ± 1.72 mmHg, heart rate 72.82 ± 1.02, post-session rtACS values: systolic pressure 129.19 ± 2.94 mmHg, diastolic pressure 82.20 ± 1.24 mmHg, heart rate 70.69 ± 0.99; means and SEM).

### Primary outcome measure

**Fig 2:**
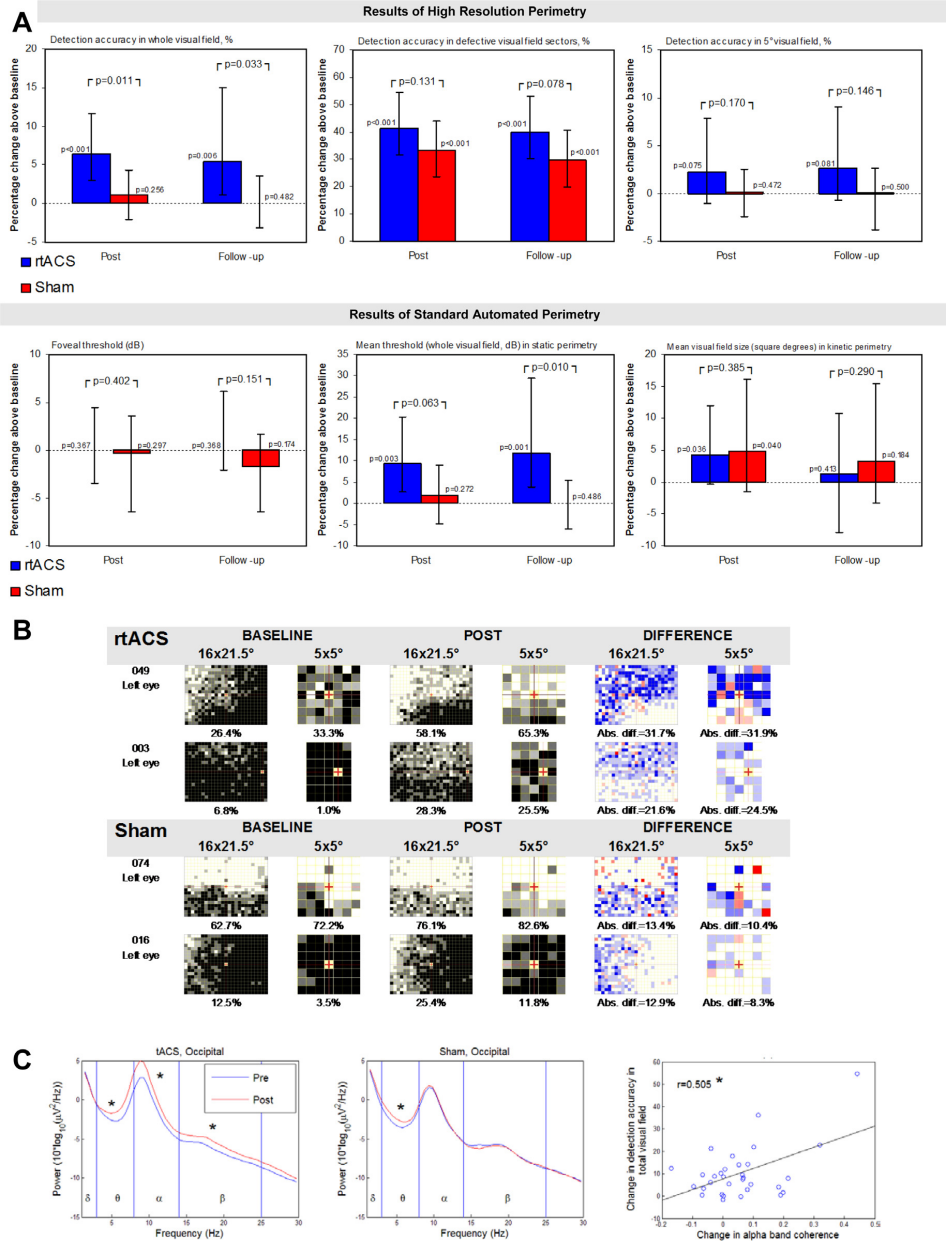
Perimetry measures and EEG. **(A)** Primary and secondary analyses of VF outcome between- and within-groups after rtACS and sham-stimulation bar charts of primary (first upper graph) and secondary parameters of VF diagnostics measured using HRP and standard-automated static and kinetic perimetry. Results are given as medians and 95%-CI. Between-group comparisons were performed according to a pre-defined hypothesis using a one-sided U-test. Within-group BASELINE vs. POST and BASELINE vs. FOLLOW-UP comparisons were calculated separately for each treatment arm using Wilcoxon matched-pairs signed rank tests. The respective p-values are reported with p<0.05 considered as significant. **(B)** Individual change in HRP VF charts at BASELINE and POST in the two best responding patients of both groups. By superimposing HRP computer campimetric VF charts of three repeated measurements, VF areas were categorized as intact (perfect stimulus detection at a given location, white spots), partially damaged/relative defect (inconsistent stimulus detection, grey spots), and absolutely impaired areas (no stimulus detected, black spots). Detection increases and decreases after intervention are shown in blue and red, respectively. The percentage improvement of the detection accuracy was comparable between the whole HRP VF 16x21.5° and the central 5° VF. **(C)** Power spectra before and after the first stimulation session. Left sub-figure: One session of tACS increased power of theta (Z = 3.583, p<0.001), alpha (t = 4.571, p<0.001) and beta bands (Z = 3.142, p = 0.002) recorded from electrode positions above the visual cortex. Middle sub-figure: After sham stimulation a significant power increase was observed for only the theta band (Z = 3.147, p = 0.002). Right sub-figure: Scatter plot showing the relation between change in alpha band coherence at the occipital area of interest and change in detection accuracy in total visual field (primary outcome measure).

Concerning the primary outcome, percent change over baseline in detection rates in VF tests, rtACS-treated patients showed significantly greater improvements than sham-treated patients (p = 0.011, Mann-Whitney U, Fig 2A). Due to the higher number of responders in the rtACS-group the mean improvement of VFs at POST was 24.0% detection accuracy after rtACS vs 2.5% after sham-treatment. The corresponding effect estimator of the median difference (BASELINE vs. POST) between treatment arms was 5.0%, CI[0.6;10.0]. While the rtACS- group exhibited significant improvements in BASELINE vs. POST comparisons of medians (Hodges-Lehmann-estimator 6.4%, CI[2.9;11.6], p<0.001 (Wilcoxon signed rank test, one-sided), the sham-group did not (1.1%, CI[-2.0;4.3]). Fig 2B depicts HRP visual charts of two patients with the greatest improvements in the primary outcome measure in both groups. Reliability parameters of HRP and eye-tracking documented excellent retinotopic reliability of the primary outcome measure, validating that improved visual detection could not be explained by altered eye movements (S2 Fig).

### Secondary outcome measures

Table 3 summarizes the results of the trial. The median between-group difference in percentage detection rate change in the defective VF did not reach statistical significance, (8.2% CI[-5.9%;22.5%, p = 0.131) because this parameter significantly increased in both groups; by 41.3% (CI[31.5; 54.3]) in the rtACS- and 33.2% (CI[23.4; 44.2]) in the sham-group (both p<0.001). Reaction time changes in HRP improved in the rtACS-group (median) by 8ms, (CI[-16;0], p = 0.023), with a trend in the sham-group of 4ms (CI[-15;2], p = 0.069); the between-group difference was not significant (p = 0.35). Other secondary outcome measures obtained at POST did not reveal significant between-group differences. For example, visual acuity did not change significantly (Table 3).

**Table 3:** Clinical parameter changes after treatment and at follow-up.

	POST vs BASELINE						FOLLOW-UP vs BASELINE					
Parameter	Within groups				Between groups		Within groups				Between groups	
	rtACS		Sham				rtACS		Sham			
	mean ±SEM	p	mean ±SEM	p	mean ±SEM	p	mean ±SEM	p	mean ±SEM	p	mean ±SEM	p
**High Resolution Perimetry**												
Detection accuracy in whole visual field, %	23.96 ±10.1	<0.001	2.53 ±2.75	0.256	21.43 ±10.46	0.011	24.98 ±11.01	0.006	0.28 ±3.34	0.482	24.70 ±11.51	0.033
Detection accuracy in defective visual field sectors, %	59.86 ±13.44	<0.001	34.83 ±5.30	<0.001	25.03 ±14.44	0.131	61.29 ±16.14	<0.001	30.72 ±5.96	<0.001	30.56 ±17.21	0.078
Detection accuracy within 5° visual field, %	63.24 ±55.67	0.075	1.13 ±3.90	0.472	62.11 ±55.80	0.170	66.74 ±51.45	0.081	-0.96 ±2.46	0.500	67.71 ±51.51	0.146
Fixation accuracy (%)	12.20 ±8.62	0.015	5.66 ±3.55	0.016	6.54 ±9.32	0.427	30.48 ±19.77	0.076	6.08 ±2.91	0.013	24.40 ±19.98	0.390
False positive reactions in %	33.75 ±9.97	0.003	22.73 ±18.10	0.237	11.01 ±20.67	0.076	50.18 ±12.92	<0.001	46.70 ±22.74	0.034	3.49 ±26.15	0.134
RT whole visual field (ms)	2.13 ±0.87	0.022	1.30 ±1.19	0.086	0.86 ±1.47	0.338	1.49 ±0.89	0.084	0.59 ±1.30	0.383	0.90 ±1.58	0.242
RT in defective visual field sectors (ms)	2.03 ±0.96	0.063	1.48 ±1.04	0.075	0.55 ±1.42	0.452	1.61 ±0.87	0.086	2.22 ±1.62	0.127	-0.60 ±1.84	0.485
RT within 5° visual field (ms)	3.46 ±1.00	0.001	2.74 ±1.43	0.013	0.72 ±1.74	0.437	3.41 ±1.27	0.006	2.44 ±1.51	0.052	0.97 ±1.97	0.383
**Static Perimetry**												
Foveal threshold (dB)	-1.72 ±4.03	0.367	0.05 ±5.80	0.297	-1.77 ±7.06	0.402	1.13 ±3.00	0.368	-7.52 ±4.22	0.174	8.65 ±5.18	0.151
Mean threshold (whole visual field, dB)	22.38 ±10.67	0.003	3.72 ±5.00	0.272	18.65 ±11.78	0.063	34.97 ±18.52	0.001	2.14 ±4.59	0.486	32.83 ±19.08	0.010
Fixation accuracy in static perimetry, %	0.93 ±3.36	0.373	2.82 ±2.62	0.129	-1.89 ±4.26	0.206	10.69 ±10.19	0.197	-4.00 ±3.62	0.205	14.70 ±10.81	0.192
**Kinetic Perimetry**												
Mean eccentricity (°)	11.62 ±6.27	0.035	6.40 ±5.13	0.063	5.22 ±8.10	0.406	2.51 ±5.45	0.426	4.47 ±4.37	0.159	-1.96 ±6.99	0.285
Mean visual field size (square degree)	27.27 ±16.44	0.036	20.47 ±15.89	0.040	6.80 ±22.90	0.385	11.23 ±11.86	0.413	9.06 ±6.90	0.184	2.17 ±13.72	0.29
**Visual acuity**												
Uncorrected near vision	-0.014 ±0.016	0.267	-0.082 ±0.020	<0.001	0.068 ±0.026	0.012	-0.066 ±0.017	0.001	-0.068 ±0.025	0.002	0.003 ±0.029	0.370
Uncorrected far vision	-0.039 ±0.023	0.067	-0.032 ±0.019	0.032	-0.007 ±0.030	0.371	-0.020 ±0.025	0.257	-0.032 ±0.019	0.064	0.012 ±0.031	0.226

For cases with binocular lesions values for both eyes were averaged. Results are given as mean and standard error mean (SEM).

After 10 days rtACS, the mean threshold in static perimetry (median change 9.3%; CI[2.6;20.3], p = 0.003) significantly increased while after sham-stimulation no significant change in threshold in static perimetry was noted (median change 1.9%; CI [-4.8%, 8.8%]). Concerning mean VF size obtained in kinetic perimetry, a clinically negligible but still significant increase was observed in both groups (median change after rtACS 4.3% [-0.3%, 11.9%]; p = 0.036, and after sham-stimulation 4.8% [-1.5%, 16.1%], p = 0.040).

Additionally, at BASELINE, POST and FOLLOW-UP, patients completed a neuropsychological test battery that included an alertness reaction time test, and a trail-making paper-pencil-test. Performance in these tests remained largely unchanged in both groups. However, a significant increase in reaction time in the alertness test was observed in the rtACS- but not in the sham-group (S1 Table). Improvements in the performance of the Trail Making Test were observed in both groups.

### Outcome assessment at 2-months Follow-Up and outcome prediction

The differences between FOLLOW-UP and BASELINE are somewhat smaller than the differences between POST and BASELINE with a persistent significant effect in the primary measure detection accuracy in HRP in terms of both within- and between-group comparisons, indicating the stability of the gains (Fig 2A). Interestingly, at FOLLOW-UP static perimetry thresholds increased beyond POST-levels in the rtACS-group and reached levels significantly higher than at BASELINE (median change 11.7%; CI[3.7;29.5]; p = 0.001) which was not observed in the sham-group. The 10.2% between-group difference at FOLLOW-UP was significant at p = 0.01 (CI[1.4%;22.8]). VF improvement as measured by kinetic perimetry did not persist after 2 months in both groups. A treatment outcome prediction model was used to predict the change in HRP visual fields based on BASELINE results [24] (S2 Fig).

### Patient-reported outcomes

In subjects with binocular vision impairment, NEI-VFQ measures of vision-related QoL indicated improved ratings of NEI-VFQ scales “VF defect and related impairments” after rtACS as well as improved ratings of “general health and mental distress” after sham. In the intervention-related questionnaire, the items “treatment was helpful”, “increased vision after treatment” at POST, and “satisfied with treatment”, “general perception” at FOLLOW-UP were more frequently answered positively after rtACS than after sham (S3 Fig).

### EEG power-spectra and functional connectivity changes

Group comparisons of the EEG-spectrum confirm that alpha- and beta but not theta power increases were significantly greater after 10 days rtACS (median change: 0.037) than after sham-stimulation (median change: 0.015), F(1,1529.605) = 6.894, p = 0.009. Unspecific power increases after 10 days sham-stimulation were only found in the theta power-band, which may reflect fatigue during the course of the experiment.

After the first rtACS session, an increase of spectral power was observed in theta, alpha and beta frequency bands in both the occipital and frontal regions. The occipital coherence change after the first stimulation session was significantly correlated with final treatment outcome (Fig 2C).

### Current flow simulation

**Fig 3:**
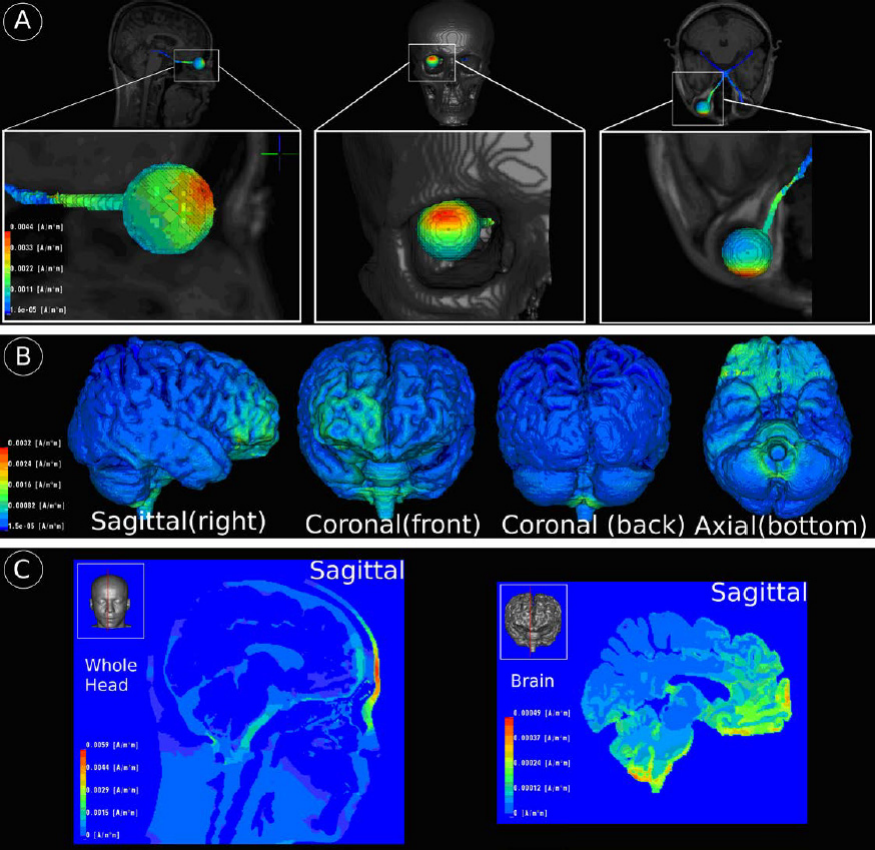
Visualization of simulated electrical fields during rtACS: current density maxima on eye/optical nerve **(A)**, brain tissue surface **(B)** and in the volume **(C)**. Although four electrodes were used for treatment, they were used only one at a time. Therefore, the current flow simulation was done with only one electrode, representing all other electrodes. **(A)** Current density maxima of about 0.0044 A/m2 can be observed on the upper part of the outer eye surface that is closest to the stimulating electrode. Furthermore, the optical nerve of the stimulated eye also receives parts of the stimulating current density magnitude as currents enter the inner skull. **(B)** Local current density maxima can be found at frontal brain regions spatially located close to the stimulating anodal electrode. **(C)** Another area of locally increased current density can be found at the brain stem and lower cerebellum.

Computer simulations of current flow in the intact brain suggest that a considerable amount of current enters the skull through the eye and optic nerve (Fig 3A), affecting brain regions close to the eye socket. Fig 3B indicates that some amount of current appears to be shunted directly through the skull into the frontal cortex as seen by current density concentrations (of approximately similar magnitude) just behind the electrode. Inside the skull, highest current densities were observed in high conductive cerebrospinal fluid (CSF) inferior to the brain stem (Fig 3C), which indicates that most of the current flows along the shortest path of lowest resistance at the skull base within CSF liquor. A major proportion of the current appears to leave the skull through the foramen magnum and down the spinal cord into the ground. There was also an increased current density magnitude at the lower cerebellum and lower brain stem (Fig 3B and 3C). In Fig 3C (left), higher current densities are also present in skin tissue close to the electrode and lower current densities at lower conducting materials such as skull tissue, internal air cavities, and tissues remotely located from current injection sites. Smaller current density magnitudes are present in gray matter and white matter tissues of the brain (Fig 3C (right)) peaking in the frontal and lower cerebral regions. The thalamus and visual cortex show only low current densities compared to other sites presumably because they are further away from the shortest path between electrodes.

## Discussion

The present randomized controlled, multi-center trial of non-invasive electrical brain stimulation documents the efficacy and safety of rtACS-treatment in patients with vision loss after optic neuropathies confirming earlier, exploratory, small-sample trials [14–16]. Repeated daily stimulation with non-invasive rtACS significantly improved detection performance in VFs with persistent effects for at least 2 months beyond the stimulation period as shown by static perimetry. Kinetic perimetry appeared to be insensitive to visual field dynamics. VF improvement in static measures was associated with increased vision-related QoL and subjective visual functioning in patients with binocular vision impairment. However, in contrast to what was seen in a previous study [14], the “objective” improvement of VFs did not correlate significantly with “subjective” measures, i.e., there was a mismatch between both levels of analysis which can be attributed to the fact that only a small proportion of subjective vision can actually be explained by “objective” VF results as previously discussed [10]. About 2 of 3 patients treated with rtACS reported being satisfied with the treatment even though only 1 of 3 patients was aware of vision improvements.

We performed computer simulation to estimate the amount of current that was being delivered to components of the visual system in a 40-yrs old male control subject. Computer simulations of rtACS current flow and estimated current density levels revealed that the stimulation current propagates through the eyeball and optic nerve to subcortical and midbrain regions with secondary stimulation at the brain stem and in cerebellar regions. Although the simulation model does not consider the extent of optic nerve damage the results provide meaningful insights indicating that most of the current affect frontal parts of the brain including eyeball and optic nerve while only low levels were found in the visual cortex. This finding is compatible with the hypothesis that cortical EEG changes after rtACS are due to “physiological” synchronous activation of retinal ganglion cells following eye and optic nerve stimulation [25]. For future studies in this area current flow simulations may help to target both safety considerations and improved stimulation paradigms.

Our findings of improved visual functions are consistent with the hypothesis that rtACS can modulate perceptual thresholds in “areas of residual vision” as previously reported in studies that also used current stimulation [12, 14–16] or behavioral vision training methods [10, 17, 26–28]. The improvement of visual functioning together with spectral power and connectivity changes in the occipital EEG alpha band [12] are consistent with the hypothesis of a retinofugal entrainment by rtACS that improves visual perception through rhythmic firing of retinal ganglion cells. Indeed, in the present study we cannot exclude that some of the effects on coherence are due to volume conduction effect. Future studies might use high-density EEG, which would allow using source reconstruction algorithms (e.g. LORETA). Such algorithms typically reduce the volume conduction problem and thus allow a more spatially precise localization of neurophysiological effects and more reliable estimation of functional coupling. As recently shown by patients with optic nerve damage who suffer from desynchronization of spatial and temporal processing deficits of their brain functional networks [12, 29] the therapeutic effects of rtACS may be mediated by re-synchronizing the brain networks, which were desynchronized by the vision loss. Because well-synchronized dynamic brain networks are critical for cognitive processing of visual information [30], we conclude that a visually deprived brain with re-synchronized functional networks can process reduced visual input more efficiently thereby activating or amplifying, residual vision even many years after the damage has occurred [10].

According to the prevailing view, tACS entrainment leads to a frequency-specific phase realignment of the endogenous oscillations with the applied alternating current with a subsequent frequency-specific power enhancement [31–35]. The beneficial functional consequence of rtACS for visual perception may possibly be explained by retinofugal entrainment and brain functional connectivity modulation [12, 16]. Another issue of interest is the specificity of the range of current frequencies used for stimulation. In future studies it will be important to systematically study the effects of specific frequencies for parameter optimization [36]. Application of the optimal frequencies might improve efficacy and will also shed light on the role of specific brain oscillations in their ability to activate residual vision.

It may be argued that visual improvements after rtACS can be explained by perceptual learning due to repeated testing. However, this is unlikely because we ascertained stable visual field baselines before starting the treatment and the primary and secondary outcome criteria did not improve to the same extent in the sham-group which was exposed to the same number of diagnostic test repetitions. Since we observed a *decrease* in RT when ACS treated patients responded to small light stimuli presented during visual field testing, another alternative explanation for improved vision outcome is a secondary effect of an increase in attention in the treatment group due to rtACS which then results in general performance improvements in vision tests as well. However, this interpretation is not supported by results of a basic alertness test. Here, we observed a general RT *increase* in response to large centrally presented stimuli as used during the alertness test (S1 Table). In other words, despite lower overall alertness, visual processing to (smaller) visual stimuli was faster. Our interpretation is that visual processing following ACS treatment may be more efficient, but the precise relationship between general alertness and modality-specific (visual) attention in patients with visual field defects requires further study.

An important question in brain stimulation studies is what constitutes an appropriate sham-condition. In prior rtACS-studies, the sham-condition was an auditory sound that mimicked current delivery [14–15]. Below phosphene-threshold stimulation was employed by others [37]. Both of these sham conditions cannot completely rule out that patients correctly guessed to which group they belonged (because of lack of phosphene experience in shams), leaving open the possibility of an expectation bias. Our sham-condition avoided this bias by using a “minimal dose” sham-condition where phosphene perceptions also occurred. However, the draw-back of this kind of sham procedure is that a “minimal dose” may actually have some, albeit small, therapeutic effects that cannot be separated from possible placebo effects of the current study design. Indeed, the sham group significantly improved in some parameters as well. The reason we selected a “minimal dose” treatment for sham controls is that this equalizes the expectations in both groups. Indeed, one would not expect that patients generate a hypothesis on their own that fewer phosphenes were less effective. However, considering that phosphenes may contribute to a placebo effect, it cannot be ruled out that patients in the rtACS-group who experienced more significant visual phenomena during stimulation might have had a greater feeling of that a powerful treatment was occurring than those who only experienced a flash once a minute.

Several open questions should be addressed in future studies. Important issues are whether non-invasive current stimulation-induced visual system changes are due to neuroplastic alteration, if they can be potentiated by vision training [38], and if rtACS can also improve vision in patients with post-chiasmatic lesions [39]. In addition, for general diagnostics low contrast visual acuity as a sensitive outcome measure and retinal nerve fiber layer thickness measurements to better define study inclusion criteria should be considered to explore altered axonal and neuronal integrity in the afferent visual pathway. While additional studies are needed to further explore the mechanisms of action, our results warrant the use of rtACS treatment in a clinical setting to activate residual vision by brain network re-synchronization, which can partially restore vision in patients with stable vision loss caused by nervous system damage.

## Appendix

### Reliability of visual field measurements

#### Reliability parameters of the primary outcome measure

The absolute change of HRP fixation accuracy and percentage of responses outside the valid response window in HRP were considered as reliability parameters. Fixation accuracy improved significantly at POST in both groups (median increase, rtACS: 1.1%, CI[0.1; 2.5], p = 0.017; sham: 1.3%, CI[0.0; 2.6], p = 0.023), with no significant difference between groups. The percentage of reactions outside the valid response window (150–1000ms) after stimulus presentation in HRP increased slightly after rtACS (0.3%, CI[0.0; 0.9], p = 0.016) and remained unchanged after sham (-0.03%, CI[-0.5; 0.2], 0.377) with a significant difference between groups (p = 0.033).

#### Eye-tracking during HRP

To determine whether changes of visual field results are related to, or could be explained by, altered fixation behavior during visual field testing, HRP visual fields were measured while eye movements were recorded with an eye-tracker (Tobii ET1750, Tobii Technology AB, Sweden) in one study center (Magdeburg). Together with reliability parameters of HRP, the eye-tracking results documented excellent retinotopic reliability of the primary outcome measure, validating that improved visual detection could not be explained by altered eye movements (such as saccades towards the blind field). Mean fixation was within the central area of 1° horizontally and 0.5° vertically for both groups at all time points (S1 Fig). Standard deviation of the mean fixation position extended up to approx. 1.5–2° of visual angle both horizontally and vertically, which is in the expected range [17]. In fact, performance change in HRP correlated negatively with changes in deviations from fixation during eye-tracked HRPs, i.e. the better the fixation, the greater the detection accuracy improvements. For both the rtACS- and sham-group significant correlations were observed between fixation performance and detection accuracy change in the whole-tested HRP visual field (r = -0.47, p<0.05), and in the defective visual field as well (r = -0.51, p<0.05).

### Treatment outcome prediction model

In order to analyze which areas of the visual field respond with improvements after rtACS, we separately analyzed different visual field states based on HRP measurements and developed a treatment outcome prediction model to predict from the BASELINE results of HRP visual fields the extent of vision restoration [24]. To this end, different visual field states (i.e. full function, partial function and absolute vision loss) were determined by superimposing repeated HRP measures to determine which visual field regions are intact, partially damaged (residual I and residual II) and absolutely impaired (absolute defect).

The difference between groups in the primary outcome measure was found to be maily caused by a reduction of the absolute (black) scotoma region in the rtACS-group. The size of the absolute defect significantly decreased after rtACS when compared to sham-treated patients (χ² = 190.201, df = 1, p<0.0001) (S2 Fig). Post-intervention changes in the intact and residual visual field did not differ between groups.

Out of a total of 12 features deemed relevant based on previous studies [24], two features of HRP visual fields at baseline were associated with visual field improvements after rtACS: greater “neighborhood activity”, i.e., the mean detection rate of all HRP test positions within a 5° radius around each detected location, and greater “residual function”, i.e. the detection rate at a given visual field position (S3 Fig). This suggests that improvements of residual vision are the key factor of vision restoration.

### Patient reported outcomes

Subjective change was evaluated with the NEI-VFQ scales “visual field defect and related impairments” and “general health and mental distress” at POST and FOLLOW-UP and compared between groups. At POST there was an improvement in NEI-VFQ scale “general health and mental distress” in the sham-group (p<0.01) but not after rtACS, with no significant difference between groups (p = 0.81). At FOLLOW-UP both groups reported subjective benefits in the NEI-VFQ scales “visual field defect and related impairments” (rtACS: p<0.01, sham: p = 0.02) and “general health and mental distress” (rtACS: p = 0.04; sham: p = 0.01), again with no significant difference between groups (p = 0.77).

Due to the possibility that patients with monocular impairment may not experience a severe reduction in vision-related QoL, an exploratory subgroup analysis of patients with binocular loss, i.e., excluding those with an intact fellow eye, was conducted. Here, rtACS-patients reported a significant increase in NEI-VFQ “visual field defect and related impairments” scale (p<0.01) at FOLLOW-UP with no significant difference between groups (p = 0.40). In the sham-group there was a significant increase in the NEI-VFQ “general health and mental distress” scale (p = 0.01) at POST with no significant difference between groups (p = 0.30).

In another intervention-related questionnaire, the items: “treatment was helpful”, “increased vision after treatment” at POST, and “satisfied with treatment”, “general perception” at FOLLOW-UP were more frequently answered positively after rtACS than after sham (S4 Fig).

## Supporting Information

S1 Fig Fixation accuracy. Fixation accuracy in eye-tracking during HRP. Eye-tracking fixation accuracy while performing a visual detection task in HRP, shown as mean vertical and horizontal fixation position in degrees of visual angle in the visual field. Fluctuations of the mean fixation positions at BASELINE, POST and FOLLOW-UP are shown as 1SD.
(PDF)

S2 Fig Visual field change according to the visual field state at BASELINE comparing rtACS- and sham-group. (PDF)

S3 Fig Relevant features predicting treatment outcome after rtACS. According to self-organizing map (SOM)-charts relevant features for prediction are “Neighborhood activity” and “Residual function”. For further explanations, see text. Improved II refers to previously defect positions where detection rate improved by 66%, improved I refers to positions where detection rate improved by 33%.
(PDF)

S4 Fig Patient-reported outcome. Patient-reported outcomes at POST and FOLLOW-UP. Results of a structured intervention-related questionnaire that also included a response category labeled “not sure”. All subjects answered the questionnaire, but “not sure” answers were given by a large number of subjects.
(PDF)

S1 File CONSORT checklist. (PDF)

S2 File Statistical analyses report. (PDF)

S1 Table Neuropsychological measures. (DOCX)

## Abbreviations

ACS: alternating current stimulation
CSF: conductive cerebrospinal fluid
HRP: high resolution perimetry
QoL: quality of life
tDCS: transcranial direct current stimulation
rtACS: repetitive transorbital alternating current stimulation
VF: visual field
