# Supplementary figures and images for: Alternating Current Stimulation for Vision Restoration after Optic Nerve Damage: A Randomized Clinical Trial
Source: PLoS One. 2016 Jun 29;11(6):e0156134. doi: 10.1371/journal.pone.0156134 (PMC4927182; doi:10.1371/journal.pone.0156134)

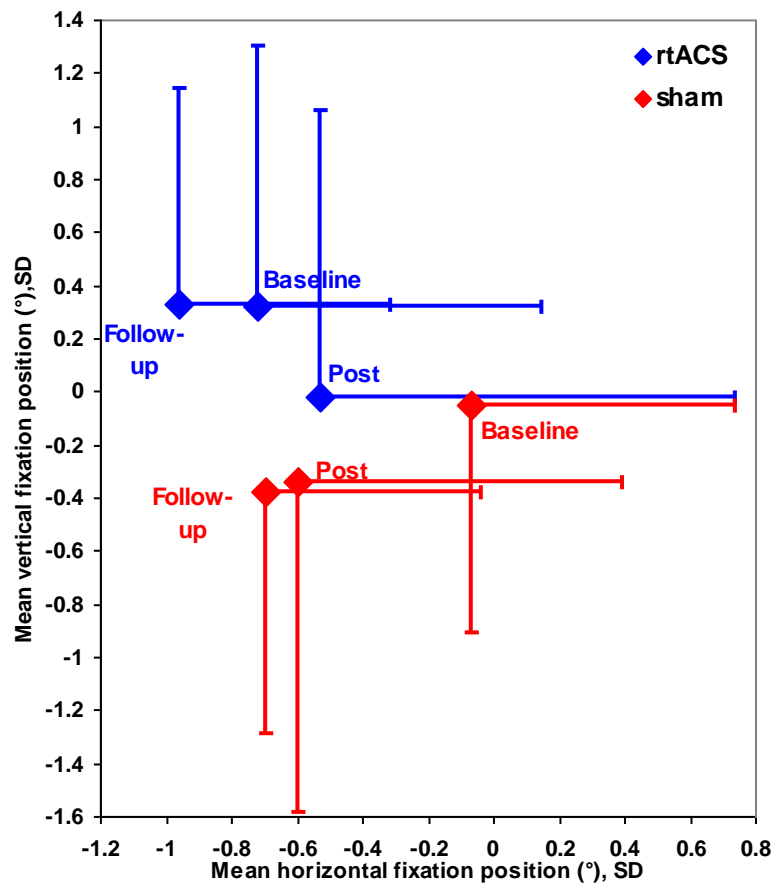

Supplement: S1 Fig — Fixation accuracy in eye-tracking during HRP. Eye-tracking fixation accuracy while performing a visual detection task in HRP, shown as mean vertical and horizontal fixation position in degrees of visual angle in the visual field. Fluctuations of the mean fixation positions at BASELINE, POST and FOLLOW-UP are shown as 1SD. (PDF) [file pone.0156134.s001.pdf]

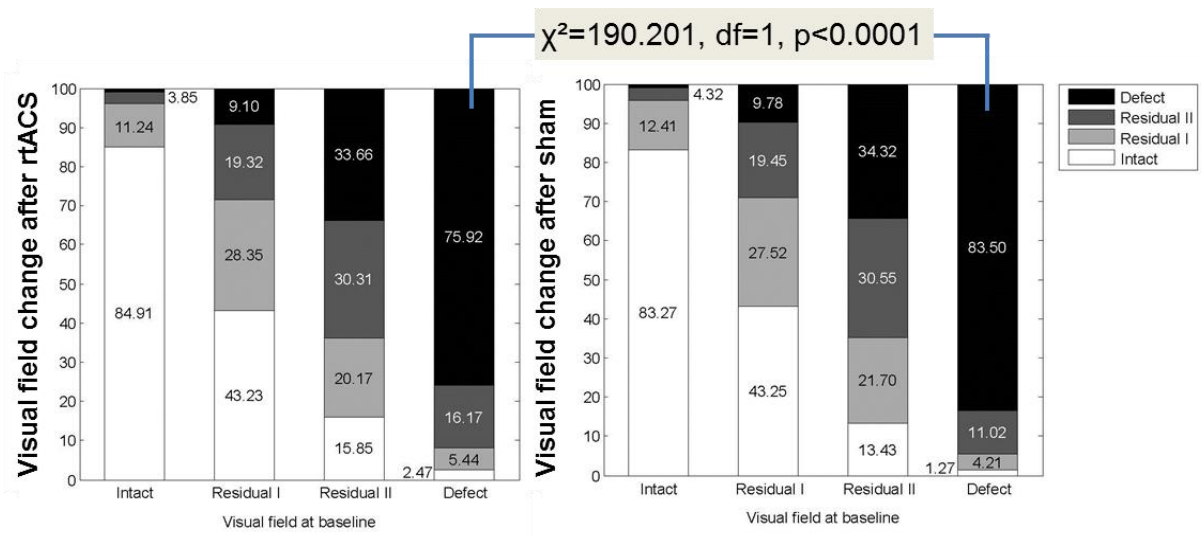

Supplement: S2 Fig — (PDF) [file pone.0156134.s002.pdf]

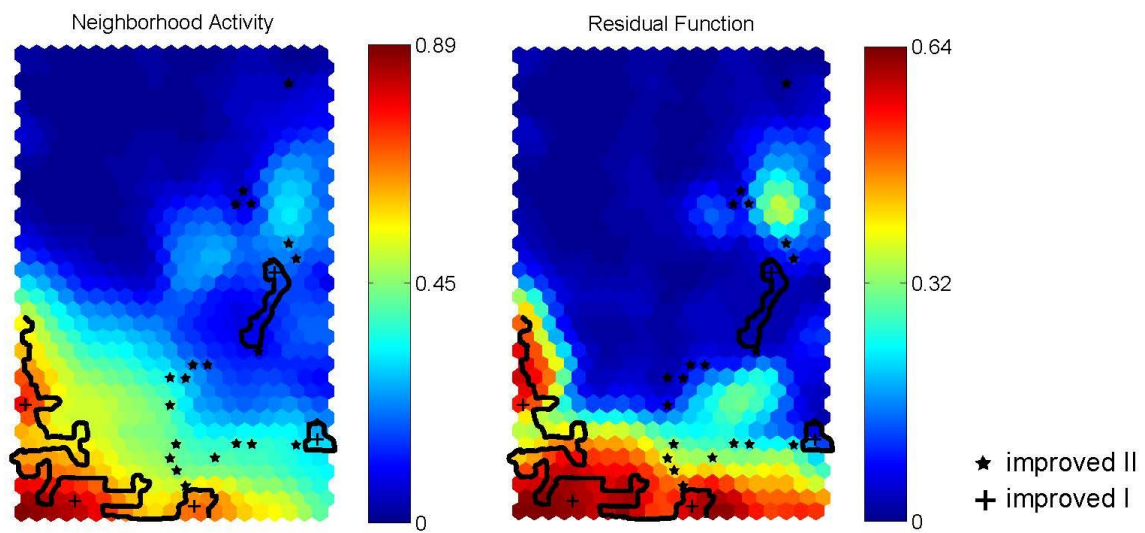

Supplement: S3 Fig — According to self-organizing map (SOM)-charts relevant features for prediction are “Neighborhood activity” and “Residual function”. For further explanations, see text. Improved II refers to previously defect positions where detection rate improved by 66%, improved I refers to positions where detection rate improved by 33%. (PDF) [file pone.0156134.s003.pdf]

### Subjective reports at POST

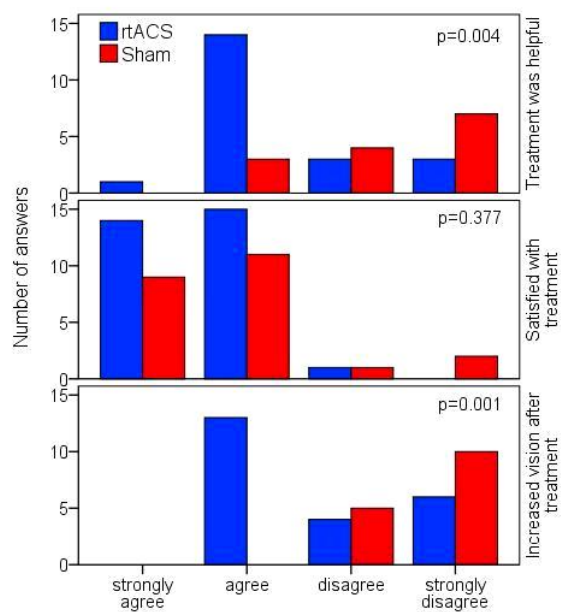

### Subjective reports at FOLLOW-UP

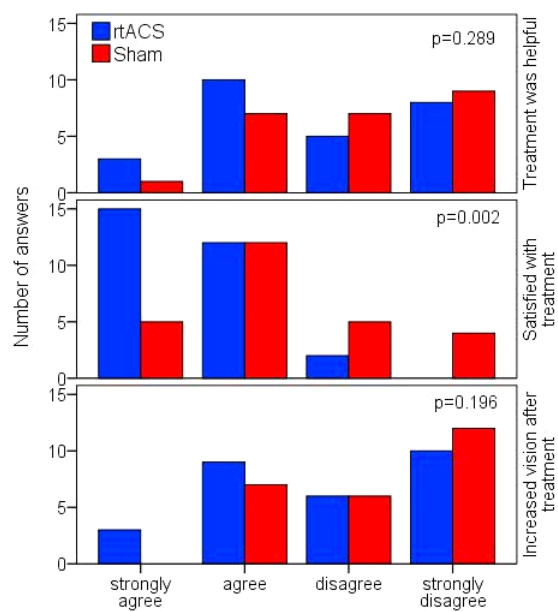

Supplement: S4 Fig — Patient-reported outcomes at POST and FOLLOW-UP. Results of a structured intervention-related questionnaire that also included a response category labeled “not sure”. All subjects answered the questionnaire, but “not sure” answers were given by a large number of subjects. (PDF) [file pone.0156134.s004.pdf]
