# Supplementary material for: Alternating Current Stimulation for Vision Restoration after Optic Nerve Damage: A Randomized Clinical Trial
Source: PLoS One. 2016 Jun 29;11(6):e0156134. doi: 10.1371/journal.pone.0156134 (PMC4927182; doi:10.1371/journal.pone.0156134)
Supplement: S2 File — (PDF) [file pone.0156134.s006.pdf]

„Multi-center study for the treatment of patients with optic neuropathy using non-invasive alternating current stimulation“

MC Study nervus opticus

Statistical Report

Date amended: 25.10.2012

State as amended: Draft 1

Principal Investigator
Prof. Bernhard A. Sabel
Institute for Medical Psychology
Leipziger Straße 44
39120 Magdeburg

Sponsor
EBS Technologies GmbH
Albert-Einstein-Ring 7
14532 Kleinmachnow

Author: Anke Lux and Prof. S. Kropf
Institute of Biometry and Medical Informatics
Leipziger Straße 44
39120 Magdeburg
Phone 0391 / 6713530
Fax 0391 / 6713536

Contents

1	General study information	3
2	Methods	3
3	Patient flow	5
4	Basic data	6
5	Analyses for study outcome	7
	High Resolution Perimetry (HRP)	7
	Static and kinetic perimetry	10
	Visual acuity	12
	Influence of the study center	14
6	Summary	15

1 General study information

The realized study is the validation of a new non-invasive electrical stimulation procedure for the treatment of optic nerve damage. In particular, an improved detection performance in a computerised campimetric visual field test (High Resolution Perimetry, HRP) is expected after electrical stimulation.

In the randomized controlled multicenter trial with double blinding a total of 90 patients with optic neuropathy had been planned to be treated and examined. In a first step, a baseline examination (at least two weeks before the initial diagnosis) was carried out. After this it was decided on the inclusion of the patient in the study. There was then a randomized division of the patients into two groups, of which one group received an electrical stimulation with stimulation electrodes placed near the eyes in trans-orbital electrode placement, the other group received a sham stimulation. The patients were extensively examined before stimulation on two days. After this initial diagnosis, each patient was stimulated on 2x5 consecutive weekdays with either non-invasive repetitive transorbital alternating current stimulation (rtACS) or sham stimulation. While the group with effective rtACS treatment (“verum”) received a supposedly “effective dose” of therapy that produced regular and long-lasting phosphenes, the sham group was treated with a “minimal dose” which produced only very short-lasting phosphenes. Immediately following completion of treatment, all initial diagnostic tests were repeated. The whole investigation and treatment period lasted about two and a half weeks. After a therapy-free interval of two months a further follow-up diagnostic was conducted.

2 Methods

The randomization of patients in verum and sham group was done with a stratified block randomization, carried out at the Institute of Medical Psychology Magdeburg centrally for all study centers. The randomization was performed using the program “Randomization In Treatment Arms” (RITA) developed and distributed by the company StatSol, a spin-off company of the University of Lübeck. The stratification considered the study center (3 levels) and the defect depth (result of the baseline measurement, 2 levels) as a potential prognostic factor. A variable block length of 4 or 6 was used, in order to obtain approximately the same group sizes for the two treatment arms.

The independent statistical analysis for this study was carried out by the Institute for Biometry and Medical Informatics at the University of Magdeburg based on the elaborated analysis plan dated on January 23rd, 2012, which was finalized prior to unblinding the trial. The analyses were done with the software IBM SPSS Statistics, Version 19. The basis of the analysis are the results of the initial, final and 2-month follow-up diagnostic of the patients participating in the study using various perimetric methods (HRP, static and kinetic perimety) and visual acuity tests.

This data were handed over to the Institute of Medical Psychology in a blinded form as three SPSS-files, after proving and comparing it independently with the analysis results of the

perimetry by a biometrics assistant. The randomization list generated and exported by the software RITA (StatSol, Lübeck) was also recorded in an SPSS file and matched with study data.

In the present biometric analysis in particular seven endpoints are regarded: the primary as well as six secondary endpoints. The primary endpoint indicates the percentage change in the stimulus detection rate at the diagnostic visit after the end of the stimulation period compared to the initial diagnostics in the visual field of the eye / eyes with visual field loss. The first secondary endpoint is the percentage change of the stimulus detection rate only related to the defective visual field of the affected eye / eyes. The second secondary endpoint indicates the average response time of the patients related of the entire visual field. The third and fourth secondary endpoints refer to the visual fields in the conventional perimetry (mean threshold in static perimetry and mean eccentricity in kinetic perimetry). The fifth and sixth endpoints consider the change in visual acuity at near and far.

The relative change (%) as the result of the initial and final diagnostics and the follow-up examination was determined separately for each endpoint (excluding visual acuity) as 100*(final value – initial value) / initial value, for the reaction time with inverted sign to express a decrease as positive change. With respect to the visual acuity, differences of the logarithmic values were considered for the statistical analysis. For patients with both eyes included, the rates were determined separately for each eye. Then initial, final and follow-up values as well as the relative and absolute changes were averaged over both eyes for use in the subsequent statistical analysis.

In addition to the above mentioned main secondary endpoints, further secondary endpoints are considered: in HRP the average reaction time of patients based on the defective visual field, the fixation accuracy and false positive reactions as parameters of reliability and in kinetic perimetry the visual field area that is enclosed by the kinetic visual field border.

Since the blinded data gave little evidence of center effects and dependencies of the improvements in the visual efficiency of the initial findings, the primary analysis was performed as a one-sided U-test for the level 0.05. The corresponding effect estimates (with 95% CI) was calculated using the Hodge-Lehmann method.
A covariance analysis for the logarithms of the relative improvements including the treatment and the study center as factors and the initial value of the percentage stimulus detection rate as a covariate was performed in a secondary analysis.
The above mentioned secondary endpoints were evaluated in an analogous way to the primary endpoint. The same is true for the analyses for the follow-up results (compared to baseline), also considered in secondary analyses.
In addition to the comparison between the two treatment arms in further secondary analysis, a comparison of stimulus detection rate between initial and final diagnostics (or follow-up) separately for each treatment group was made, including Wilcoxon matched-pairs signed rank tests and corresponding Hodges-Lehmann confidence intervals.

3 Patient flow

In total, 98 patients participated in the study. Of these, 51 were assigned to the verum group, 47 to the sham group. Overall, there was an exclusion of 13 patients before or during the stimulation period (verum group: 4, sham group: 9). Of the remaining 85 patients, three patients were excluded from the analysis (verum group: 2, sham group: 1) because of lack of residual vision which is contradicting the according inclusion criterion. Thus, 82 patients remained in the analysis related to the comparison of final to initial diagnostics. 2 of 82 patients (from verum group) were lost in the follow-up investigation. For further information on the exclusion see the flow chart.

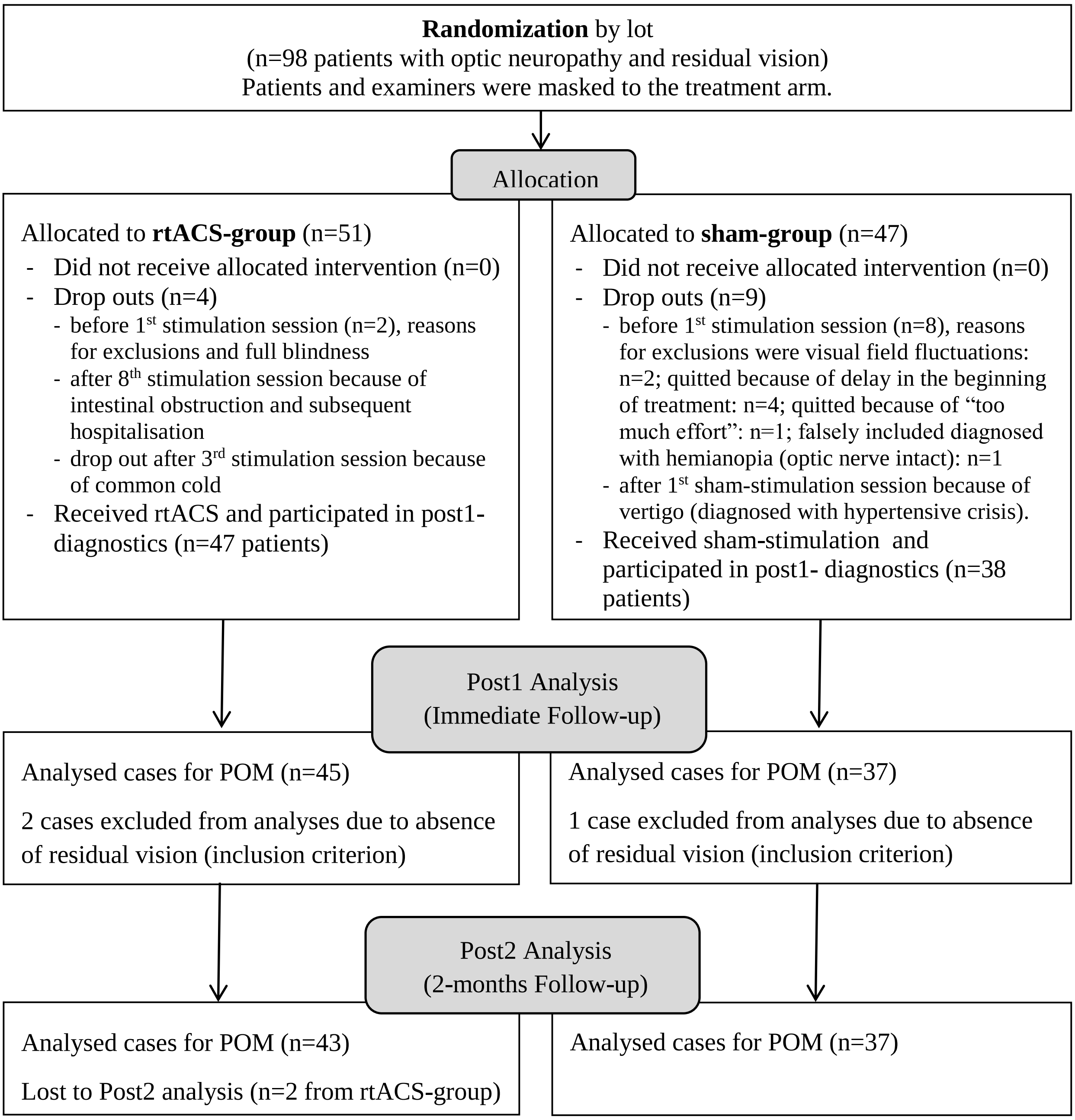

Figure 1: Patient flow

4 Basic data

The total study population (as used in the primary analysis) consisted of 47 men (57.3%) and 35 women (42.7%) aged 23 to 83 years, the average age was 59.1 ± 13.1 years.
44 of 82 patients (53.7%) were included with both eyes, the remaining 38 patients (46.4%) were included with one eye only. In patients with one impaired eye, the other eye was either intact in 31 patients (37.9%) or blind in 7 patients (8.5%). The lesion age was in 11 patients (13.4%) between 6 and 12 months, in 7 patients (8.5%) between 1 and 2 years, and in 63 patients (76.8%) the lesion was older than 2 years.
The most frequent cause / type of lesion was glaucoma which occurred in 33 cases (40.2%), followed by AION in 16 cases (19.5%) and NAION in 15 cases (18.3%). In 9 patients (11.0%) there were two causal diagnoses.
In the result of the baseline measurement a classification of patients according to high and low defect depth was made. In 55 patients (67.1%) a high defect depth and in 27 patients (32.9%) a low defect depth was observed. Table 1 provides an overview of these demographic and clinical parameters, according to the two treatment arms.

Table 1: Patient characteristics

		**Treatment arm**			
		**verum**		**sham**	
Age (years, $\bar{x} \pm SD$)		57.8 ± 14.2		60.7 ± 11.6	
		N	%	n	%
Sex	Male	32	71.1	15	40.5
	Female	13	28.9	22	59.5
Lesion	Binocular	27	60.0	17	45.9
	monocular (one eye intact)	14	31.1	17	45.9
	monocular (one eye blind)	4	8.9	3	8.1
Lesion age OD	6 - 12 months	5	13.9	2	7.4
	1 - 2 years	3	8.3	0	0.0
	> 2 years	28	77.8	25	92.6
Lesion age OS	6 - 12 months	3	8.3	4	14.8
	1 - 2 years	6	16.7	2	7.4
	> 2 years	27	75.0	21	77.8
Type of lesion	glaucoma	16	35.6	17	45.9
	AION	12	26.7	4	10.8
	NAION	7	15.6	8	21.6
Defect depth	below 30% (high)	32	71.1	23	62.2
	above 30% (low)	13	28.9	14	37.8

The results of the baseline (initial) values the HRP, static and kinetic perimetry, and visual acuity are summarized in Table 2. The vision investigation revealed information on visual acuity in near and far. On the one hand, all examined patients were considered, on the other hand those were excluded who could only recognize a hand movement (corresponding to

logMAR = 3). The observed differences between the two groups in the values of the initial diagnostics were not statistically significant (Wilcoxon-Mann-Whitney U test, two-sided).

Table 2: Initial diagnostics

	**Treatment arm**		
	**verum**	**sham**	**p**
**High-resolution perimetry,** $\bar{x} \pm SEM$			
Detection accuracy in whole visual field (%)	44.94 ± 3.81	52.70 ± 4.28	0.142
Detection accuracy in defective visual field sectors (%)	21.98 ± 2.00	25.95 ± 2.51	0.228
Fixation accuracy (%)	83.03 ± 3.59	87.28 ± 2.65	0.586
False positive reactions (%)	3.46 ± 0.69	3.65 ± 0.95	0.155
RT whole visual field (ms)	537.74 ± 13.78	516.39 ± 11.74	0.394
RT in HRP defective visual field sectors (ms)	559.05 ± 10.67	539.47 ± 10.44	0.261
**Standard automated perimetry,** $\bar{x} \pm SEM$			
Foveal threshold (dB) in static perimetry	19.97 ± 1.50	23.60 ± 1.39	0.113
Mean threshold (whole visual field, dB) in static perimetry	10.18 ± 0.93	11.99 ± 1.15	0.320
Fixation accuracy in static perimetry, %	78.88 ± 4.57	87.86 ± 3.32	0.259
Mean eccentricity (degree) in kinetic perimetry	42.73 ± 2.56	42.48 ± 2.93	0.899
Mean visual field size (square degree) in kinetic perimetry	7170.31 ± 551.94	7061.64 ± 685.96	0.817
**Visual acuity (logMAR),** $\bar{x} \pm SEM$			
Uncorrected near vision	1.04 ± 0.12	0.92 ± 0.09	0.970
Uncorrected far vision	1.25 ± 0.17	0.75 ± 0.13	0.072
**Visual acuity (logMAR),** $\bar{x} \pm SEM$ **(Exclusion of patients with value 3)**			
Uncorrected near vision (n=77)	0.85 ± 0.09	0.86 ± 0.07	0.267
Uncorrected far vision (n=69)	0.68 ± 0.11	0.62 ± 0.10	0.708

Even though differences are not significant, they underline the need to consider the relative values (final value / initial value) for the main analysis.

5 Analyses for study outcome

High Resolution Perimetry (HRP)

Concerning the primary outcome criterion, the percentage change of the “detection rate in the total visual field” in the final versus initial diagnostics using the high resolution perimetry

method (HRP) was calculated separately for both treatment arms (using one-sample Wilcoxon signed rank test against reference value 0, one-sided). Here, a significant increase in detection performance was observed only in the verum group (p <0.001). The Hodges-Lehmann estimator for the median increase as the related effect estimator was 6.4% with 95% confidence interval (CI) of [2.9%, 11.6%]. The mean increase amounted at 24.0%. In the sham group, the increase was not significant (*p* = 0.256, one-sided) at an estimated median increase of 1.1% (95% confidence interval [-2.0%; 4.3%]) respectively a mean increase of 2.5%.
Regarding the endpoint "detection rate in the defective visual field", both treatment arms showed a significant increase (*p* < 0,001) with an estimated median increase of 41.3% (95% CI [31.5%; 54.3%]) in the verum group and a median increase of 33.2% (95% CI [23.4%; 44.2%]) in the sham group. The mean increases achieve 59.9% in the verum group and 34.8% in the sham group. Reaction times significantly decreased in the verum group (*p* = 0.022, one-sided; estimation of median decrease of 1.5% [0%; 2.9%]). In the sham group, however, no significant decrease of the reaction time was measured (*p* = 0.086, one-sided; estimation of median decrease of 0.7% [-0.6%; 3.1%]).
Regarding the fixation accuracy, in both treatment groups there is a significant increase (in the verum group with p = 0.015 and estimated median increase of 1.4% [0.1%, 4.0%]; in the sham group with p = 0.016 and a median increase of 1.6% [0.2%, 3.1%]). There is also an increase in false-positive reactions. This is significant in the verum group (p = 0.016, one-sided) at an estimated median increase of 25.6% (95% CI [6.8%, 50.4%]). In the sham group, however, there is an estimated median increase of 6.0% (95% CI [11.5%, 29.0%]) which is not significant (p = 0.237, one-sided).

According to the analysis plan, the primary analysis was directed to the comparison of percentage changes in detection rates between the two treatment arms (verum vs. sham) in a one-sided Mann-Whitney U test. The test reveals a significant difference (*p* = 0.011) in favor of the verum treatment for the total visual field (primary endpoint). For the corresponding effect estimator (median group difference between the two treatments) the Hodges-Lehmann estimator was 5.0% with 95% CI [0.6%, 10.0%]. For the secondary endpoint "relative change of stimulus detection rate in defective visual field", the median difference in the percentage change between both treatment arms is also positive (in favor of verum) with an estimate of 8.2% and the 95% CI [-5.9%; 22.5%] but not significant (*p* = 0.131, one-sided U test). The percentage decrease of reaction time in the verum group is according to the Hodges-Lehmann estimator 0.6% larger than in the sham group (95% CI [-1.3%; 2.6%]). This difference is not significant (*p* = 0.338, one-sided U test). Regarding the fixation accuracy the verum group shows a by 0.2% lower median increase than the sham group (95% CI [-2.0%, 2.1%]), which is not significant (p = 0.427, one-sided U test). At the false positive reactions the verum group shows a by 20.0% larger median increase than the sham group (95% CI [-5.8%, 43.0%]), which is, however, not significant (p = 0.076, one-sided U test).

Due to high improvements of several patients in the verum group, the *mean* group difference is even larger than the above mentioned *median* difference. The mean percentage improvement

of the stimulus detection rates in the total visual field in the verum group is 21.4% larger than in the sham group (95% CI [0.4%; 42.4%]). The corresponding mean improvement in the affected visual field in the verum group is 25.0% larger than in the sham group (95% CI [-3.9%, 54.0%]), the relative decrease of reaction time is 0.8% larger (95% CI [-2.1%, 3.8%]). Furthermore, the increase of fixation accuracy in the verum group is 6.5% larger (95% CI [-12.1, 25.2]) and the increase of false-positive reactions is 11.0% larger (95% CI [-30.4, 52.4]). The previously described results are summarized in Table 3.

Table 3: High Resolution Perimetry, final versus initial diagnostics

**Parameter**	**Within groups change 1**					**Between groups change 1**	
	**Total sample size**	**rtACS-group**		**Sham-group**			
		$\bar{x}\pm$ SEM	p	$\bar{x}\pm$ SEM	p	$\bar{x}\pm$ SEM	p
Detection accuracy in whole visual field (%)	82	23.96 ± 10.10	<0.001	2.53 ± 2.75	0.256	21.43 ± 10.46	0.011
Detection accuracy in defective visual field sectors (%)	82	59.86 ± 13.44	<0.001	34.83 ± 5.30	<0.001	25.03 ± 14.44	0.131
Fixation accuracy (%)	82	12.20 ± 8.62	0.015	5.66 ± 3.55	0.016	6.54 ± 9.32	0.427
False positive reactions (%)	82	33.75 ± 9.97	0.003	22.73 ± 18.10	0.237	11.01 ± 20.67	0.076
RT whole visual field (ms)	82	2.13 ± 0.87	0.022	1.30 ± 1.19	0.086	0.86 ± 1.47	0.338
RT in HRP defective visual field sectors (ms)	82	2.03 ± 0.96	0.063	1.48 ± 1.04	0.075	0.55 ± 1.42	0.452

Thus, a significant difference between both groups was found in the primary outcome measure. But there were no significant differences in any of the secondary outcome criteria.

The comparison of follow-up versus initial diagnostics shows similar results, both related to the percentage change in the stimulus detection rate separately for treatment groups, as well as to the comparison of the between groups difference in the percentage improvements of stimulus detection rates. The differences of follow-up vs. initial diagnostics are somewhat smaller than the differences of post treatment diagnostics vs. initial diagnostics. Excluded from this statement is the increase in false-positive reactions. This is significant in both groups with p < 0.001 and a median increase of 40.7% (95% CI [19.8%, 66.2%]) in the verum group, and p= 0.034 and a median increase of 19.2% (95% CI [-1.2%, 47.2%]) in the sham group. The results are summarized in Table 4.

Table 4: High Resolution Perimetry, follow-up vs. initial diagnostics

Parameter		Within groups change 2				Between groups change 2	
	Total sample size	rtACS-group		Sham-group			
		x̄ ± SEM	p	x̄ ± SEM	p	x̄ ± SEM	p
Detection accuracy in whole visual field (%)	80	24.98 ± 11.01	0.006	0.28 ± 3.34	0.482	24.70 ± 11.51	0.033
Detection accuracy in defective visual field sectors (%)	80	61.29 ± 16.14	<0.001	30.72 ± 5.96	<0.001	30.56 ± 17.21	0.078
Fixation accuracy (%)	80	30.48 ± 19.77	0.076	6.08 ± 2.91	0.013	24.40 ± 19.98	0.390
False positive reactions (%)	80	50.18 ± 12.92	<0.001	46.70 ± 22.74	0.034	3.49 ± 26.15	0.134
RT whole visual field (ms)	80	1.49 ± 0.89	0.084	0.59 ± 1.30	0.383	0.90 ± 1.58	0.242
RT in HRP defective visual field sectors (ms)	80	1.61 ± 0.87	0.086	2.22 ± 1.62	0.127	-0.60 ± 1.84	0.485

Static and kinetic perimetry

Further visual field investigations were carried out using conventional perimetry. This included the mean threshold of the differential light sensitivity (in the static perimetry) and the mean eccentricity of the external visual field boundary and the associated area (in kinetic perimetry).

Looking at the change in the mean threshold of final versus initial diagnostics separately for both treatment groups (using the Wilcoxon pair difference test against reference value 0, one-sided), there is a significant increase in differential light sensitivity in the verum group (p = 0.003). For the median increase as corresponding effect estimator the Hodges-Lehmann estimator was 9.3% with 95% confidence interval [2.6%, 20.3%]. The mean increase was 22.4%. In the sham group, the increase was not significant (p = 0.272) with an estimated median increase of 1.9% (95% confidence interval [-4.8%, 8.8%]) and a mean increase of 3.7%. With respect to the mean eccentricity, there is also a significant improvement of size of the visual field in the verum group (p = 0.035, one-sided) with an estimated median increase of 2.1% (95% CI [-0.1%, 8, 5%]). In the sham group, there is a tendency for significant improvement (p = 0.063, one-sided; estimate of the median increase 2.5% [-1.5%, 8.1%]).
For the area enclosed by the kinetic visual field border, associated with the mean eccentricity, there are similar results. A significant increase of visual field area was observed in both groups,

in the verum group (p = 0.036, estimation of median increase 4.3% [-0.3%, 11.9%]) and in the sham group (p = 0.040, estimation of median increase 4.8% [-1.5%, 16.1%]).

To compare the differences in the changes of the mean threshold and eccentricity between the two treatment arms the one-sided U-test was used. Regarding the change in the differential light sensitivity the median difference tends to be significant in favor of the verum treatment (p = 0.063). For the corresponding effect estimator (median group difference between the two treatments) the Hodges-Lehmann estimate is 6.6% and the 95% confidence interval [1.9%, 17.2%]. For the change of size of the visual field the median difference in the percentage change between both treatment arms is positive (in favor of verum) with an estimate of 0.4% and the 95% CI [-4.1%; 5.7%] but not significant (*p* = 0.406, one-sided). For the associated area the verum group shows a by 0.6% larger median increase than the sham group (95% CI [-8.7%, 8.8%]), which is also not significant (p = 0.385, one-sided).
The results for the static and kinetic perimetry are summarized in Table 5.

Table 5: Perimetry, final versus initial diagnostics

**Parameter**	**Within groups change 1**					**Between groups change 1**	
	**Total sample size**	**rtACS-group**		**Sham-group**			
		$\bar{x} \pm$ SEM	p	$\bar{x} \pm$ SEM	p	$\bar{x} \pm$ SEM	p
**Static Perimetry**							
Foveal threshold (dB)	79	-1.72 ± 4.03	0.367	0.05 ± 5.80	0.297	-1.77 ± 7.06	0.402
Mean threshold (whole visual field, dB) in static perimetry	80	22.38 ± 10.67	0.003	3.72 ± 5.00	0.272	18.65 ± 11.78	0.063
Fixation accuracy in static perimetry, %	76	0.93 ± 3.36	0.373	2.82 ± 2.62	0.129	-1.89 ± 4.26	0.206
**Kinetic Perimetry**							
Mean eccentricity (°) in kinetic perimetry	72	11.62 ± 6.27	0.035	6.40 ± 5.13	0.063	5.22 ± 8.10	0.406
Mean visual field size (square degree) in kinetic perimetry	72	27.27 ± 16.44	0.036	20.47 ± 15.89	0.040	6.80 ± 22.90	0.385

When considering follow-up versus initial diagnostics, separately for each treatment group as well as compared between the two treatment groups, the results for the static perimetry are again similar as those for the comparison of final and initial diagnostics. The differences are partially even more pronounced. With respect to the differential light sensitivity in the verum group, a median increase of 11.7% (95% CI [3.7%, 29.5%], p = 0.001) and a mean increase of 35.0% is obtained. This increase is significantly larger than in the sham group (p = 0.010) with a by 10.2% larger median increase (95% CI [1.4%, 22.8%]).

No significant differences are found in the kinetic perimetry when comparing follow-up versus initial diagnosis separately in both treatment groups, though there are positive increases (verum group: p = 0.426, median increase 0.5% [-5.2%, 4.5%]; sham group: p = 0.159, median increase 1.9% [-1.7%, 7.3%]).
The results for comparison of follow-up versus initial diagnosis can be found in the Table 6.

Table 6: Perimetry, follow-up versus initial diagnostics

Parameter	Within groups change 2					Between groups change 2	
	Total sample size	rtACS-group		Sham-group			
		x̄± SEM	p	x̄± SEM	p	x̄± SEM	p
**Static Perimetry**							
Foveal threshold (dB)	72	1.13 ± 3.00	0.368	-7.52 ± 4.22	0.174	8.65 ± 5.18	0.151
Mean threshold (whole visual field, dB) in static perimetry	71	34.97 ± 18.52	0.001	2.14 ± 4.59	0.486	32.83 ± 19.08	0.010
Fixation accuracy in static perimetry, %	73	10.69 ± 10.19	0.197	-4.00 ± 3.62	0.205	14.70 ± 10.81	0.192
**Kinetic Perimetry**							
Mean eccentricity (°) in kinetic perimetry	65	2.51 ± 5.45	0.426	4.47 ± 4.37	0.159	-1.96 ± 6.99	0.285
Mean visual field size (square degree) in kinetic perimetry	65	11.23 ± 11.86	0.413	9.06 ± 6.90	0.184	2.17 ± 13.72	0.290

Visual acuity

In addition to the various visual field investigations, visual acuity values were obtained. Particularly, the uncorrected visual acuity at near and far vision was measured. The obtained values were recorded as logMAR values.

Regarding the change in mean visual acuity at near vision of final versus initial diagnostics separately for both treatment arms (using the Wilcoxon pair difference test against reference value 0, one-sided), there was a (not significant) decrease in logMAR values (p = 0.134) in the verum group, where decreasing logMAR values correspond to an improvement in visual acuity. The Hodges-Lehmann estimator for the median decrease is -0.025 with a 95% confidence interval [-0.050, 0.005]. In the sham group, the improvement is significant (p <0.001) at an estimated median decrease of logMAR values of -0.060 (95% confidence interval [-0.110, -0.040]). The change in mean visual acuity at far vision in the verum group, did not reach significance (p = 0.074) with an estimated median decrease of -0 (95% CI

[-0.0045, 0]) is found. In the sham group, the improvement is again significant (p = 0.040 with a median decrease of -0.035 [-0.067, 0]).

The differences in the changes in visual acuity between the two treatment arms were compared again with one-sided U-test. In addition, the corresponding effect estimator (median group difference between the two treatment arms) was calculated using the Hodges-Lehmann method. As regards the change in visual acuity in near vision, the verum group shows a by 0.050 lower change than the sham group (95% CI [0, 0.100]), which is significant (p = 0.044). Regarding the change in visual acuity in far vision, the verum group shows a by 0 lower change than the sham group (95% CI [0, 0.060]), which is not significant (p = 0.268).
For the remaining patients after exclusion of those with logMAR = 3, the results are essentially similar. The results for the entire and the reduced group of patients are summarized in Table 7.

Table 7: Visual acuity, final versus initial diagnostics

**Parameter**	**Within groups change 1**					**Between groups change 1**	
	**Total sample size**	**rtACS-group**		**Sham-group**			
		$\bar{x}$ ± SEM	p	$\bar{x}$ ± SEM	p	$\bar{x}$ ± SEM	p
**Visual acuity**							
Uncorrected near vision	82	-0.095 ± 0.062	0.134	-0.080 ± 0.020	<0.001	-0.015 ± 0.065	0.044
Uncorrected far vision	82	-0.050 ± 0.044	0.074	-0.027 ± 0.043	0.040	-0.023 ± 0.061	0.268
**Visual acuity** (exclusion of patients with value 3)							
Uncorrected near vision	76	-0.014 ± 0.016	0.267	-0.082 ± 0.020	<0.001	0.068 ± 0.026	0.012
Uncorrected far vision	68	-0.039 ± 0.023	0.067	-0.032 ± 0.019	0.032	-0.007 ± 0.030	0.371

In the comparison of follow-up versus initial diagnostics, there are some different results, both when considering the change in mean visual acuity separately for treatment groups, as well as in comparison of the differences in the changes in visual acuity between the two treatment groups. Regarding the change in mean visual acuity in near vision, both treatment groups show a significant improvement in visual acuity (p = 0.001) with an estimated median decrease -0.072 [-0.100, -0.025] for the verum group and an estimated median decrease of -0.075 [-0.125, -0.030] in the sham group. With regard to the change in mean visual acuity at far vision, there is in none of the two groups a significant improvement (verum group: p = 0.363, median change 0 [-0.030, 0.027]; sham group: p = 0.491, median change 0 [-0.045, 0.030]). In comparison between the two treatment groups, there is neither in near vision nor in far vision a significant difference. For the reduced group of patients comparable results are found. The results for the comparison of follow-up compared versus initial diagnostics are summarized in Table 8.

Table 8: Visual acuity, follow-up versus initial diagnostics

**Parameter**	**Within groups change 2**					**Between groups change 2**	
	**Total sample size**	**rtACS-group**		**Sham-group**			
		$\bar{x}\pm$ SEM	p	$\bar{x}\pm$ SEM	p	$\bar{x}\pm$ SEM	p
**Visual acuity**							
Uncorrected near vision	78	-0.144 ± 0.067	0.001	-0.082 ± 0.035	0.001	-0.062 ± 0.075	0.383
Uncorrected far vision	78	-0.030 ± 0.089	0.363	0.128 ± 0.092	0.491	-0.158 ± 0.128	0.451
**Visual acuity** (exclusion of patients with value 3)							
Uncorrected near vision	72	-0.066 ± 0.017	0.001	-0.068 ± 0.025	0.002	0.003 ± 0.029	0.370
Uncorrected far vision	62	-0.020 ± 0.025	0.257	-0.032 ± 0.019	0.064	0.012 ± 0.031	0.226

Influence of the study centers

As secondary analysis for the comparison of therapies, a covariance analysis with simultaneous consideration of the study center and the respective initial value of the primary and secondary endpoints was used. In order to achieve a better normal approximation, these analyses were based on the logarithm of the relative increase of the endpoints, i.e. on ln(final value / initial value). Additionally to the two fixed factors treatment arm and study center, their interaction was included in the analysis. The original values of the endpoints (non-logarithmic) are used for the baseline values as covariables. The calculations are executed in the same way for all endpoints considered here: the detection rate in the entire as in the defective visual field, the mean reaction time in the entire visual field, the mean threshold in the static perimetry well as the mean eccentricity in the kinetic perimetry and finally the visual acuity at near and far.

Table 9 shows the results (*p*-values) for these tests. The indicated *p*-values are two-sided as usual in analysis of variance. They show that no significant differences occur between the study centers, neither as main factor nor as interaction. The baseline values have a significant influence on the change for some of the secondary variables. The improvement in reaction time is larger for patients with large baseline values. For the mean eccentricity and for the mean visual field size in kinetic perimetry, large baseline values correspond to a smaller improvement. In these analyses with a larger number of factors or covariables included, the treatment effects are expectedly less significant than in the primary analyses. Thus the effect of

the primary endpoint shortly failed to show significance (p = 0.069), whereas the other endpoints are far from significance.

Table 9: Results of covariance analyses

Factor / covariable		Treatment group	Study center	Treatment group * Study center	Baseline value
p-value for factor / covariable	Detection accuracy in whole visual field	0.069	0.771	0.608	0.259
	Detection accuracy in defective visual field sectors	0.139	0.915	0.699	0.627
	Reaction time in whole visual field	0.789	0.707	0.204	0.038
	Mean threshold in static perimetry	0.412	0.829	0.513	0.223
	Mean eccentricity in kinetic perimetry	0.639	0.550	0.706	<0.001
	Mean visual field size in kinetic perimetry	0.768	0.733	0.703	0.002
	Uncorrected near vision	0.989	0.675	0.901	0.035
	Uncorrected far vision	0.761	0.741	0.185	0.210

6 Summary

The study based on a double-blind, randomized, sham-controlled clinical trial verifies that the treatment with non-invasive alternating current stimulation (ACS) is effective in improving visual functions in patients with optic nerve injury when this is assessed with a computer-based high-resolution perimetric procedure (HRP). With a mean increase of 24%, ACS-treated patients showed significantly better improvements in the primary endpoint in this test **(**stimulus detection rate in the entire visual field) compared to patients of the control group (sham) with an increase of only 2.5% during treatment. The change in the first of the six secondary criteria (stimulus detection rate in the defective visual field) is indicative of a positive effect of ACS compared to sham stimulation. Though it was not significant (due to the high variance), the difference in the mean relative change between both treatment arms is large with a mean improvement of 59.9% in ACS group compared to 34.8% in the sham group. But for each group, a significant improvement in these endpoints could be found. The improvement in the entire visual field was lower than in the visual field defect in both groups. The mean reaction time in the entire visual field as an additional secondary endpoint has decreased more in the ACS group with -2.03% than in the sham group with -1.48%. The decrease was significant in

the ACS group but not in the sham group. However, the between groups comparison was not significant.

In addition, in conventional perimetry an improvement of the visual fields was found in the verum group. The ACS group achieved a significant increase of the differential light sensitivity in the static perimetry of 22.4%, whereas the control group achieved only an increase of 3.7% (mean values). Here, the difference between the two groups was almost significant. The ACS group also showed a significant increase of size of the visual field (in the kinetic perimetry) of 11.6% compared to the sham group, which showed an increase of 6.4% (mean values). Although the improvement in ACS was more pronounced, the difference between the treatment groups was not significant, arguing against a treatment effect.
An improvement in visual acuity in near or far vision under ACS compared to the control group could not be shown.

In the follow-up, the ACS-treated patients showed for the primary endpoint (stimulus detection rate in the entire visual field) a mean increase of 24.5%. This improvement was significantly larger than for patients of the control group with an increase of only 0.3%.
Regarding the stimulus detection rate in the defect visual field, the mean improvement of 61.3% for ACS compared with 30.7% for Sham shows an advantage of the verum group, but this failed significance. The improvement in the entire visual field was again lower than that in the defective visual field. The mean reaction time in the entire visual field has even further decreased compared to the diagnostics at the end of treatment (verum group: -1.5%; sham group -0.6%). The change was also not significant, however.
In static perimetry at follow-up there was still an improvement for the ACS group compared to baseline. The ACS group showed a significant increase of the differential light sensitivity of 35.0% compared to the control group with a mean increase of 2.1%. The difference between the two groups was significant. In the kinetic perimetry the mean increase of size of the visual field in the ACS group (2.5%) was significantly lower than in the comparison of final versus initial diagnosis, and even lower than in the sham group (4.4%).
An improvement in visual acuity in near or far vision compared between ACS- and control group could not be shown in the follow-up.

Magdeburg, November 7th 2012

Anke Lux Prof. Dr. Siegfried Kropf
