# Supplementary material for: Alternating Current Stimulation for Vision Restoration after Optic Nerve Damage: A Randomized Clinical Trial
Source: PLoS One. 2016 Jun 29;11(6):e0156134. doi: 10.1371/journal.pone.0156134 (PMC4927182; doi:10.1371/journal.pone.0156134)
Supplement: S1 Table — (DOCX) [file pone.0156134.s007.docx]

**S1 Table. Neuropsychological measures.**

|  | **rtACS**  **POST – BASELINE** | **Sham**  **POST – BASELINE** | **rtACS**  **FOLLOW-UP – BASELINE** | **Sham**  **FOLLOW-UP – BASELINE** |
| --- | --- | --- | --- | --- |
| Alertness, reaction time without acoustic cue (ms) | 14.76 +/- 29.58  **  (n=44) | 8.43 +/- 29.42  (n=37) | 14.13 +/- 46.03  (n=42) | 8.49 +/- 6.88  (n=37) |
| Alertness, reaction time without acoustic cue, percentile rank | -9.27 +/- 17.83  ***  (n=44) | -2.87 +/- 19.93  (n=37) | -7.52 +/- 2.89  *  (n=42) | -2.89 +/- 3.56  (n=37) |
| Alertness, reaction time with acoustic cue (ms) | 15.24 +/- 65.55  (n=44) | 6.57 +/- 40.26  (n=37) | 25.62 +/- 12.95  (n=42) | 0.68 +/- 7.24  (n=37) |
| Alertness, reaction time with acoustic cue, percentile rank | -2.30 +/- 15.54  (n=44) | -4.51 +/- 19.43  (n=37) | -5.16 +/- 2.61  (n=42) | -3.97 +/- 3.60  (n=37) |
| Phasic alertness, percentile rank | 9.02 +/- 28.38  *  (n=44) | -1.22 +/- 28.71  (n=37) | -5.04 +/- 5.02  (n=42) | 1.97 +/- 3.93  (n=37) |
| Trail making test, performance, time in s | 2.40 +/- 5.32  **  (n=40) | 4.64 +/- 7.52  ***  (n=36) | 3.65 +/- 0.77  ***  (n=39) | 5.51 +/- 2.66  *  (n=37) |

p<0.05 *, p<0.01 **, p<0.001 ***.
